# Body mass index and gestational weight gain in migrant women by birth regions compared with Swedish-born women: A registry linkage study of 0.5 million pregnancies

**DOI:** 10.1371/journal.pone.0241319

**Published:** 2020-10-29

**Authors:** Pontus Henriksson, Johanna Sandborg, Marie Blomberg, Paulina Nowicka, Kerstin Petersson, Marcus Bendtsen, Magdalena Rosell, Marie Löf

**Affiliations:** 1 Department of Health, Medicine and Caring Sciences, Linköping University, Linköping, Sweden; 2 Department of Biosciences and Nutrition, Karolinska Institutet, Huddinge, Sweden; 3 Department of Obstetrics and Gynecology, Linköping University, Linköping, Sweden; 4 Department of Clinical and Experimental Medicine, Linköping University, Linköping, Sweden; 5 Department of Food Studies, Nutrition and Dietetics, Uppsala University, Uppsala, Sweden; 6 Department of Clinical Sciences, Obstetrics and Gynecology, Umeå University, Umeå, Sweden; McMaster University, CANADA

## Abstract

**Introduction:**

Women migrating to high-income countries may have increased risks of adverse pregnancy outcomes as compared with native-born women. However, little is known whether migrant women are more likely to have unhealthy body mass index (BMI) or gestational weight gain (GWG), which is of importance considering the well-established links between unhealthy BMI and GWG with adverse pregnancy outcomes. Hence, the aim of the study was to examine the prevalence and estimate odds ratios (ORs) of underweight and obesity in the first trimester as well as inadequate and excessive GWG across birth regions in migrant (first-generation) and Swedish-born women in a population-based sample of pregnant women in Sweden.

**Methods:**

This population-based study included 535 609 pregnancies from the Swedish Pregnancy Register between the years 2010–2018. This register has a coverage of approximately 90% and includes data on body weight, height, birth country and educational attainment. BMI in the first trimester of pregnancy was classified as underweight, normal weight, overweight and obesity whereas GWG was classified as inadequate, adequate and excessive according to the recommendations from the National Academy of Medicine, USA. BMI and GWG were examined according to 7 birth regions and the 100 individual birth countries. Adjusted ORs of underweight, obesity as well as inadequate or excessive GWG by birth regions were estimated using multinomial logistic regression.

**Results:**

There were large disparities in unhealthy BMI and GWG across birth regions. For instance, women born in North Africa and Middle East and Sub-Saharan Africa had 1.40 (95% CI 1.35–1.44) and 2.13 (95% CI 2.03–2.23) higher odds of obesity compared with women born in Sweden. However, women born in Sub-Saharan Africa had also considerably higher odds of underweight (OR, 2.93 [95% CI 2.70–3.18]) and inadequate GWG (OR, 1.97 [95% CI 1.87–2.07]). The limitations of the study include the lack of a validated measure of acculturation and that the study only had data on first-generation migration.

**Conclusions:**

The large differences across the 7 regions and 100 countries highlights the importance of considering birth region and country-specific risks of unhealthy BMI and GWG in first-generation migrant women. Furthermore, inadequate GWG was common among pregnant first-generation migrant women, especially in women born in Sub-Saharan Africa, which demonstrates the need to promote adequate GWG, not only the avoidance of excessive GWG. Thus, our findings also indicate that additional support and interventions may be needed for first-generation migrant women from certain birth regions and countries in order to tackle the observed disparities in unhealthy BMI and GWG. Although further studies are needed, our results are useful for identifying groups of women at increased risk of unhealthy BMI and weight gain during pregnancy.

## Introduction

Obesity is a major global public health issue, both in the general population [1] as well as in pregnant women [2]. It is well-established that obesity and excessive gestational weight gain (GWG), as well as underweight and inadequate GWG, are associated with adverse pregnancy outcomes [2–5]. For instance, obesity and/or excessive GWG have been linked to a greater risk of cesarean deliveries, perinatal mortality, and later maternal and offspring obesity [2, 4, 5]. Also, underweight and/or inadequate GWG have been shown to be associated with higher risks for adverse pregnancy outcomes such as low birth weight and preterm birth [2, 5].

International migrants are a significant and growing population in many European countries and migrant health, including reproductive health, has been identified as an important public health priority [6, 7]. The health of migrants is influenced by numerous factors including the pre-migration status as well as environmental factors, socioeconomic factors and health behaviors in the receiving country [6, 8, 9]. Importantly, several studies have concluded that migrant women, may have an increased risk of obesity, as well as adverse pregnancy outcomes [8–11], as compared with native-born women. According to data, particularly from the USA, the prevalence of unhealthy BMI and GWG differs between ethnic groups [12–15] (e.g. white, black, Hispanic, Asian). Little is however known whether similar differences exist across birth regions in women who have migrated to a receiving country (first-generation migrants) and women born in that country (i.e. native-born). In particular, data from European populations are scarce, which is of importance considering the large differences in migration patterns between Europe and the USA [11]. Considering the well-established links between modifiable risk factors such as BMI and GWG with adverse maternal and child health outcomes [2–5], both in the short and long term, further knowledge regarding weight status and GWG of migrant women is warranted.

The aim of this study was therefore to examine the prevalence and estimate odds ratios of underweight and obesity in the first trimester as well as inadequate and excessive GWG according to birth regions in migrant (first-generation) and Swedish-born women in a large (n = 535 609) population-based sample of pregnant women in Sweden. Given the large sample size, we were able to compare BMI and GWG in women born in Sweden to first-generation migrant women born in all seven super-regions [16] (i.e. 1. Central Europe, Eastern Europe and Central Asia; 2. High income countries excluding Sweden; 3. Latin America and Caribbean; 4. North Africa and Middle East; 5. South Asia; 6. Southeast Asia and East Asia; 7. Sub-Saharan Africa) as well as 100 individual countries.

## Materials and methods

### Study design and study population

This population-based study utilized data from the Swedish Pregnancy Register, which covers approximately 90% of all births in Sweden [17]. A validation of this registry showed that the weight and height measurements had good coverage (≥ 96.0%) and agreement (≥ 92.9%, r = 0.98) with medical records [18]. The study has received approval by the Regional Ethical Review Board, Stockholm, Sweden (2018/656-31) which determined that informed consent from the study participants was not required given the nature of this registry study. PH had access to a database from the Swedish Pregnancy Register with the study variables to derive the analytic sample. The study is reported according to the Reporting of studies Conducted using Observational Routinely-collected Data (RECORD) statement (S1 File).

We utilized data from the Swedish Pregnancy Register between the years 2010–2018. In total, data for 841 503 pregnancies from women aged 15–55 years with a singleton pregnancy was available. Of these pregnancies, 86 590 had missing data of birth country and another 134 057 did not have a recorded first visit in maternity care during the first trimester (up to 13 weeks of gestation). Furthermore, there were another 17 829 pregnancies with missing data (n = 17 001) or implausible data (< 15.0 or > 70.0 kg/m^2^, n = 828) for BMI. Finally, 67 418 had missing data for covariates (i.e. educational attainment and parity) and thus 535 609 pregnancies were included in the analytic sample for the aims regarding BMI in the first trimester of pregnancy. Women included in the analyses were comparable to women who were excluded in terms of average BMI at the first visit in maternity care (24.7 vs. 25.1 kg/m^2^), age (30.8 vs. 31.0 years), the proportion of women with a university degree (52.6% vs. 49.3%) and multiparity (56.1% vs. 58.8%).

The exact gestational age for the recording of weight at the end of pregnancy is available in the Swedish Pregnancy Register since 2014. Thus, for the analyses of GWG, we included the 270 044 pregnancies which had a registered weight and gestational week in late pregnancy (34.0–43.0 gestational weeks). The women with these 270 044 pregnancies were comparable to the women not included in the analyses due to lack of GWG data (n = 265 565) regarding average age (30.8 vs. 30.8 years), BMI (24.7 vs. 24.7 kg/m^2^) and to the proportion of women with a university degree (53.1% vs. 51.6%) and multiparity (57.0% vs. 55.2%).

### Study variables

Data on the women´s own birth country, age, parity and educational attainment (no education or elementary school, high school or university) was reported by the women at the first visit in antenatal care. Sweden was treated as one region and the remaining countries were grouped into seven super-regions as described in the Global Burden of Disease (GBD) study [16]: 1) Central Europe, Eastern Europe and Central Asia, 2) High income countries (Sweden not included), 3) Latin America and Caribbean, 4) North Africa and Middle East, 5) South Asia, 6) Southeast Asia and East Asia, and 7) Sub-Saharan Africa. Educational attainment was classified as low (no education or elementary school), middle (high school, i.e. 12 years) and high (university). Body weight and height were measured at the first antenatal visit. Body weight is also measured continuously throughout pregnancy at the offered routine visits in antenatal care in which the last registered body weight in pregnancy is automatically transferred into the Swedish Pregnancy Register and was utilized in the current study. BMI was calculated as body weight (kg) divided by height (m) squared and classified into underweight (BMI < 18.5 kg/m^2^), normal weight (BMI = 18.5–24.9 kg/m^2^), overweight (BMI = 25.0–29.9 kg/m^2^) and obesity (BMI ≥ 30.0 kg/m^2^). GWG was calculated as body weight in late pregnancy minus body weight at the first antenatal visit. We then utilized the GWG recommendations by the National Academy of Medicine [3], USA, (formerly Institute of Medicine) (i.e. underweight: 12.5–18.0 kg; normal weight: 11.5–16.0 kg; overweight: 7.0–11.5 kg; obesity: 5.0–9.0 kg). To account for the fact that women have their last registered weight before labor, we individually tailored these GWG cut-offs for the recommended weekly weight gain in the second and third trimester [19] (i.e. underweight: 0.51 kg/week; normal weight: 0.42 kg/week; overweight: 0.28 kg/week; obesity: 0.22 kg/week) so they would be appropriate for the gestational age in which their last body weight was measured (see S3 File for more information).

### Statistical analysis

Multinomial logistic regression was utilized to calculate odds ratios (ORs), with their 95% confidence intervals (CIs), for obesity, overweight and underweight (normal weight = reference) as well as for inadequate and excessive GWG (adequate GWG = reference). Firstly, we examined associations of birth country with BMI and GWG using regression models with i) basic adjustment (age, [parity 0 vs. ≥1] and gestational age at first antenatal care visit) ii) basic adjustment and maternal educational attainment (low/middle/high). Basic adjustments (i.e. age, parity, gestational age at first visit) were selected since they differed between birth regions and may be linked to the study outcomes and thus may be relevant covariates. For these models, Swedish women were used as the reference category. Thereafter, we examined the combined associations of birth region and educational attainment (i.e. low/middle vs. high educational attainment) and these models were also adjusted by the aforementioned basic covariates. For these models, the reference category was Swedish women with high educational attainment. We also fitted unadjusted models for our main analysis although these results were very similar to the models with the basic adjustment (see S1 and S2 Tables). The original analysis plan is presented in S2 File. There were some changes in the analyses in response to the reviewers’ comments. First, we only included those with a measured BMI in the first trimester of pregnancy (instead of up to gestational week 16). Second, we modified our GWG calculations by tailoring the recommended GWG (instead of adjusting the weight gain of each woman). Finally, we fitted multinomial regression models (instead of binominal regression as outlined in the original analysis).

## Results

### Odds ratios of obesity and underweight by birth regions

**Fig 1:**
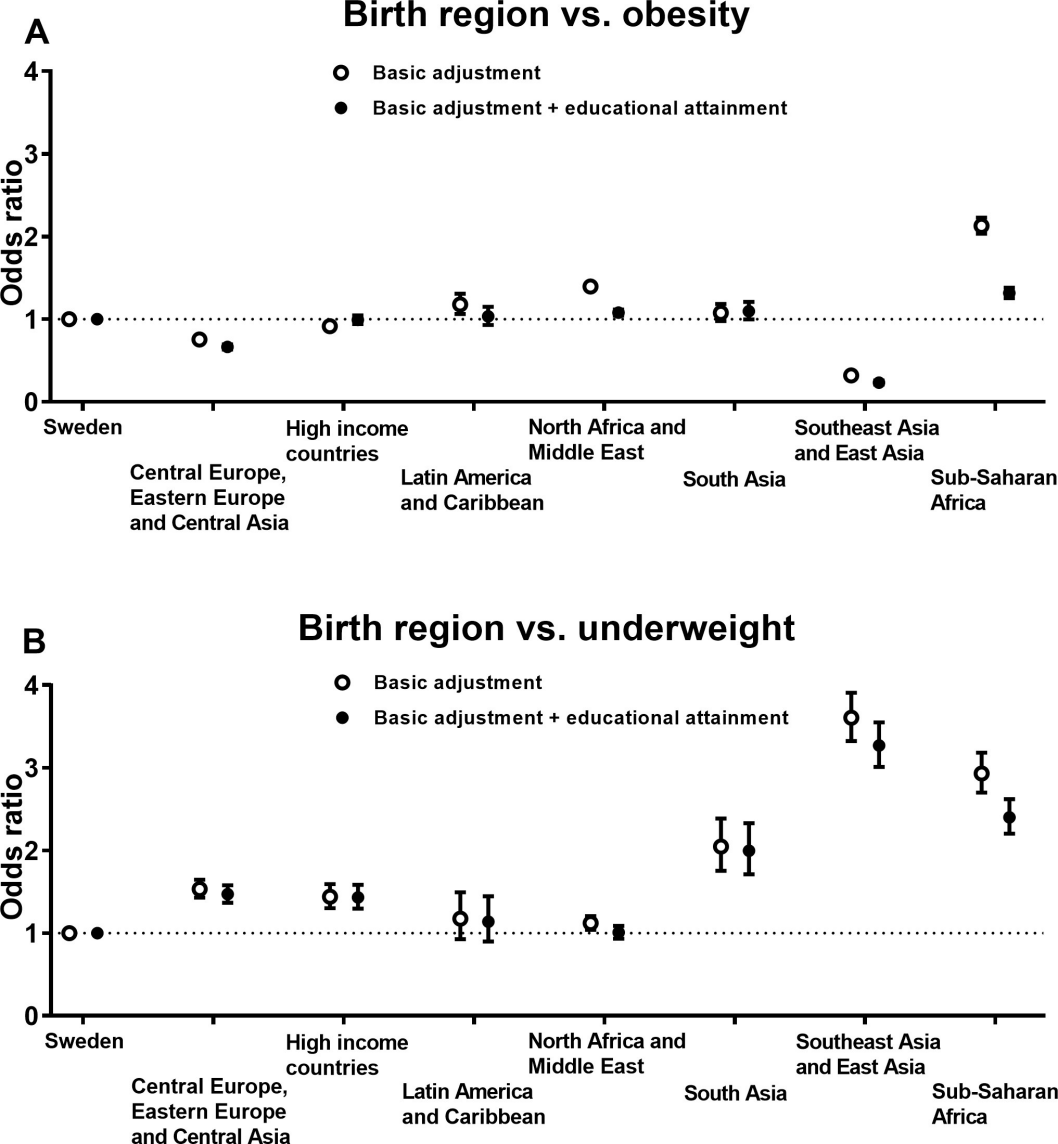
Odds ratios of (**A**) obesity and (**B**) underweight in the first trimester of pregnancy by birth regions. Multinomial logistic regression was used to estimate odds ratios with 95% confidence intervals (women born in Sweden was the reference group). Basic adjustments in the analyses were age, parity and gestational age at first antenatal care visit. Models were also further adjusted for educational attainment.

Descriptive data of the 535 609 pregnancies stratified by birth regions are presented in Table 1. Adjusted ORs of obesity (Fig 1A) and underweight (Fig 1B) in pregnancy by birth regions are presented in Fig 1 (all results in detail including estimates of overweight are available in S1 Table). Women born in Sub-Saharan Africa (OR, 2.13 [95% CI, 2.03 to 2.23]) and North Africa and Middle East (OR, 1.40 [95% CI, 1.35 to 1.44]) had statistically significantly greater odds of obesity in the basic model (adjusted for age, parity and gestational age) compared with women born in Sweden. However, these associations became considerably weaker when adjusting for educational attainment, i.e. Sub-Saharan Africa (OR, 1.32 [95% CI, 1.25 to 1.38]) and North Africa and Middle East (OR, 1.08 [95% CI, 1.04 to 1.12]).

**Table 1:** Descriptive data of the women in the study.

Characteristics	Sweden	Central Europe, Eastern Europe and Central Asia^1^	High income countries^2^	Latin America and Caribbean^3^	North Africa and Middle East^4^	South Asia^5^	Southeast Asia and East Asia^6^	Sub-Saharan Africa^7^
**n (%)**	431 978 (80.7%)	24 045 (4.5%)	14 526 (2.7%)	3 274 (0.6%)	35 089 (6.6%)	4 337 (0.8%)	8 547 (1.6%)	13 813 (2.6%)
**Age (years)**	30.7±5.0	30.6±5.0	32.8±4.8	32.2±5.2	30.2±5.5	30.6±4.4	32.1±4.9	30.4±5.6
**Primiparous**	45.1%	42.1%	44.0%	42.8%	34.8%	46.3%	44.3%	31.1%
**Educational attainment**								
Low	4.1%	12.1%	4.2%	8.5%	28.1%	11.2%	22.7%	47.6%
Middle	40.7%	41.4%	25.1%	42.2%	38.3%	28.9%	36.3%	37.3%
High	55.2%	46.4%	70.7%	49.3%	33.6%	59.9%	40.9%	15.1%
**Gestational age at first weight (wk)**	8.7±2.1	8.8±2.1	8.7±2.0	8.7±2.0	8.5±2.0	8.6±2.0	8.8±2.0	9.2±2.1
**Gestational age at last weight (wk)**	38.0±1.7	37.9±1.6	37.9±1.6	37.7±1.5	37.8±1.6	37.7±1.6	37.7±1.5	38.1±1.7
**BMI (kg/m**^**2**^**)**	24.7±4.7	24.1±4.4	24.4±4.7	25.1±4.5	25.5±4.6	24.7±4.5	22.7±3.7	25.9±5.2
**BMI-category**								
Underweight	2.3%	3.6%	2.9%	2.2%	2.2%	4.1%	8.3%	5.0%
Normal weight	60.6%	63.3%	62.9%	55.1%	49.8%	54.4%	69.9%	43.3%
Overweight	24.4%	22.9%	22.4%	29.2%	32.6%	29.1%	17.1%	31.7%
Obesity	12.7%	10.1%	11.9%	13.5%	15.3%	12.3%	4.6%	20.0%
**GWG**^8^ **(kg)**	13.6±5.1	14.4±5.3	13.0±4.8	12.5±4.8	13.2±5.4	12.1±5.0	13.1±4.4	10.3±5.5
**GWG-category**^9^								
Inadequate	17.5%	14.5%	21.1%	21.9%	17.4%	24.9%	20.8%	34.7%
Adequate	35.1%	33.2%	37.9%	35.6%	32.5%	34.9%	43.1%	34.9%
Excessive	47.4%	52.4%	41.0%	42.5%	50.1%	40.2%	36.2%	30.4%

Data are presented as mean ± standard deviations or as %.

BMI, body mass index; GWG, gestational weight gain.

^1^ Included regions/countries were: Central Asia (Armenia, Azerbaijan, Georgia, Kazakhstan, Kyrgyzstan, Mongolia, Tajikistan, Turkmenistan, Uzbekistan); Central Europe (Albania, Bosnia and Herzegovina, Bulgaria, Croatia, Czech Republic, Hungary, Macedonia, Montenegro, Poland, Romania, Serbia, Slovakia, Slovenia); Eastern Europe (Belarus, Estonia, Latvia, Lithuania, Moldova, Russia, Ukraine).

^2^ Included countries were: Australasia (Australia, New Zealand); High-income Asia Pacific (Brunei, Japan, Singapore, South Korea); High-income North America (Canada, Greenland, USA); Southern Latin America (Argentina, Chile, Uruguay); Western Europe (Andorra, Austria, Belgium, Cyprus, Denmark, Finland, France, Germany, Greece, Iceland, Ireland, Israel, Italy, Luxembourg, Malta, Netherlands, Norway, Portugal, Spain, Switzerland, UK).

^3^ Included regions/countries were: Andean Latin America (Bolivia, Ecuador, Peru); Caribbean (Antigua and Barbuda, The Bahamas, Barbados, Belize, Bermuda, Cuba, Dominica, Dominican Republic, Grenada, Guyana, Haiti, Jamaica, Puerto Rico, Saint Lucia, Saint Vincent and the Grenadines, Suriname, Trinidad and Tobago, Virgin Islands); Central Latin America (Colombia, Costa Rica, El Salvador, Guatemala, Honduras, Mexico, Nicaragua, Panama, Venezuela); Tropical Latin America (Brazil, Paraguay).

^4^ Included regions/countries were: Afghanistan, Algeria, Bahrain, Egypt, Iran, Iraq, Jordan, Kuwait, Lebanon, Libya, Morocco, Oman, Palestine, Qatar, Saudi Arabia, Sudan, Syria, Tunisia, Turkey, United Arab Emirates, Yemen.

^5^ Included regions/countries were: Bangladesh, Bhutan, India, Nepal, Pakistan.

^6^ Included regions/countries were: East Asia (China, North Korea, Taiwan (province of China)); Southeast Asia (Cambodia, Indonesia, Laos, Malaysia, Maldives, Mauritius, Myanmar, Philippines, Sri Lanka, Seychelles, Thailand, Timor-Leste, Vietnam). Oceania was not included due too few observations.

^7^ Included regions/countries were: Central Sub-Saharan Africa (Angola, Central African Republic, Democratic Republic of the Congo, Equatorial Guinea, Gabon); Eastern Sub-Saharan Africa (Burundi, Comoros, Djibouti, Eritrea, Ethiopia, Kenya, Madagascar, Malawi, Mozambique, Rwanda, Somalia, South Sudan, Tanzania, Uganda, Zambia); Southern Sub-Saharan Africa (Botswana, Lesotho, Namibia, South Africa, Swaziland (eSwatini), Zimbabwe); Western Sub-Saharan Africa (Benin, Burkina Faso, Cameroon, Cape Verde, Chad, Côte d’Ivoire, The Gambia, Ghana, Guinea, Guinea-Bissau, Liberia, Mali, Mauritania, Niger, Nigeria, São Tomé and Príncipe, Senegal, Sierra Leone, Togo).

^8^ Number of pregnancies with GWG data were as follow; Sweden (n = 209 454), Central Europe, Eastern Europe and Central Asia (n = 14 277), High income countries (n = 7 902), Latin America and Caribbean (n = 1 843), North Africa and Middle East (n = 20 757), South Asia.

(n = 2 510), Southeast Asia and East Asia (n = 4 621), Sub-Saharan Africa (n = 8 680).

^9^ GWG as below (inadequate), within (adequate) or above (excessive) the National Academy of Medicine (formerly Institute of Medicine) recommendations.

Women born in Southeast Asia and East Asia had considerably greater odds of underweight compared with women born in Sweden (OR, 3.61 [95% CI, 3.33 to 3.91 in the basic adjusted model]. Correspondingly, women born in Sub-Saharan Africa (OR, 2.93 [95% CI, 2.70 to 3.18]) and South Asia (OR, 2.05 [95% CI, 1.76 to 2.39]) had more than twice the odds of underweight than women born in Sweden in the basic model. Generally, the odds for underweight were relatively unaffected by further adjustment for educational attainment.

### Odds ratios of excessive and inadequate gestational weight gain by birth regions

**Fig 2:**
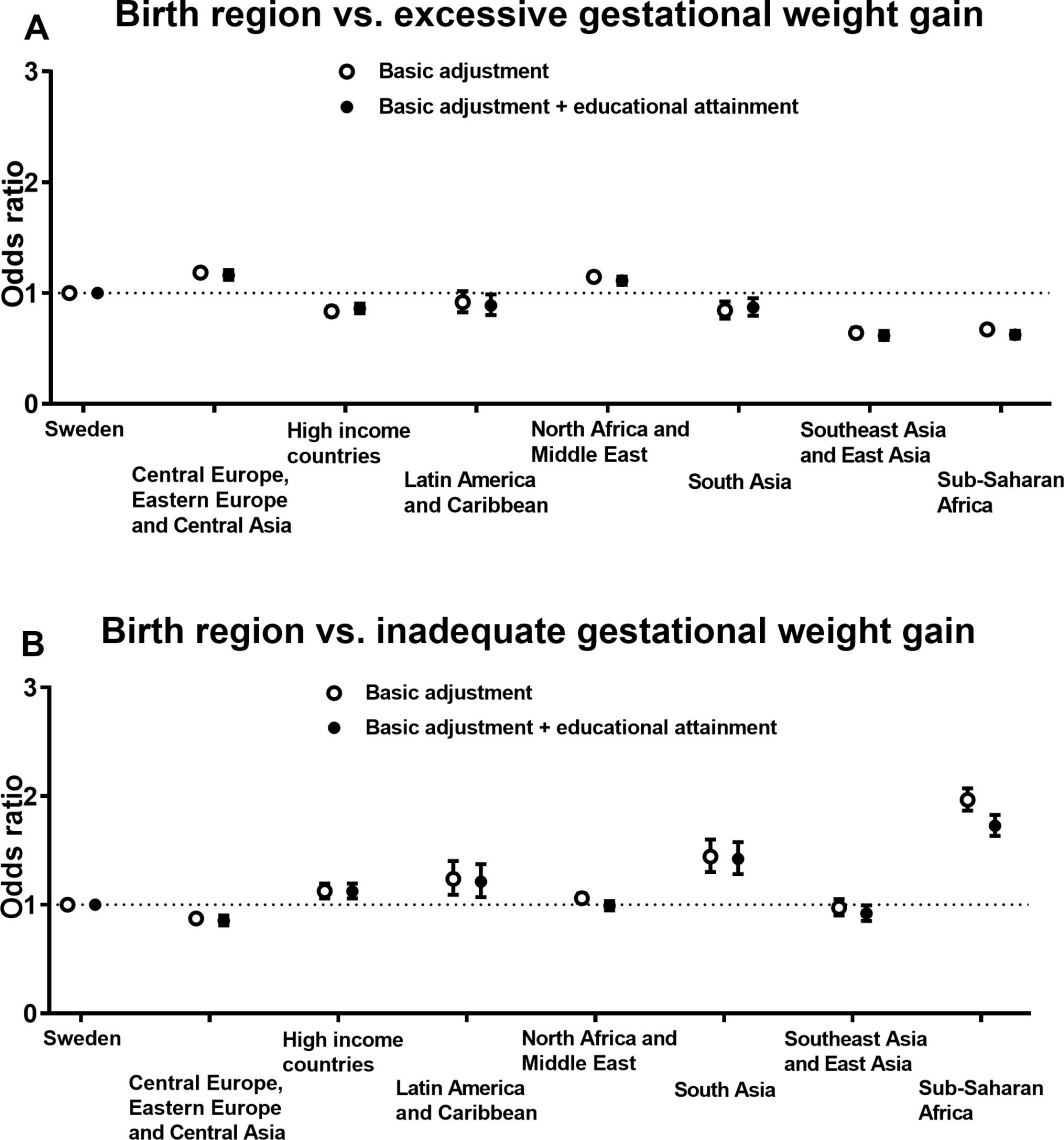
Odds ratios of (**A**) excessive and (**B**) inadequate gestational weight gain by birth regions. Multinomial logistic regression was used to estimate odds ratios with 95% confidence intervals (women born in Sweden was the reference group). Basic adjustments in the analyses were age, parity and gestational age at first antenatal care visit. Models were also further adjusted for educational attainment.

Fig 2 shows the ORs of excessive (Fig 2A) and inadequate GWG (Fig 2B) by birth region (detailed data in S2 Table). Women born in Central Europe, Eastern Europe and Central Asia (OR, 1.18 [95% CI, 1.14 to 1.23]) or North Africa and Middle East (OR, 1.15 [95% CI, 1.11 to 1.18]) had slightly greater odds of excessive GWG as compared with women born in Sweden. Conversely, women born in Sub-Saharan Africa (OR, 0.67 [95% CI, 0.64 to 0.71]), Southeast Asia and East Asia (OR, 0.64 [95% CI, 0.60 to 0.68]) had the lowest odds of excessive GWG, after basic adjustments. The estimates were generally unaffected by further adjustments for educational attainment.

Furthermore, women born in Sub-Saharan Africa were considerably more likely to have inadequate GWG compared with women born in Sweden (OR, 1.97 [95% CI, 1.87 to 2.07]) in the basic model. Generally, the associations were only marginally attenuated after further adjustments for educational attainment.

### Odds ratios of unhealthy BMI and gestational weight gain by combinations of birth regions and educational attainment

**Fig 3:**
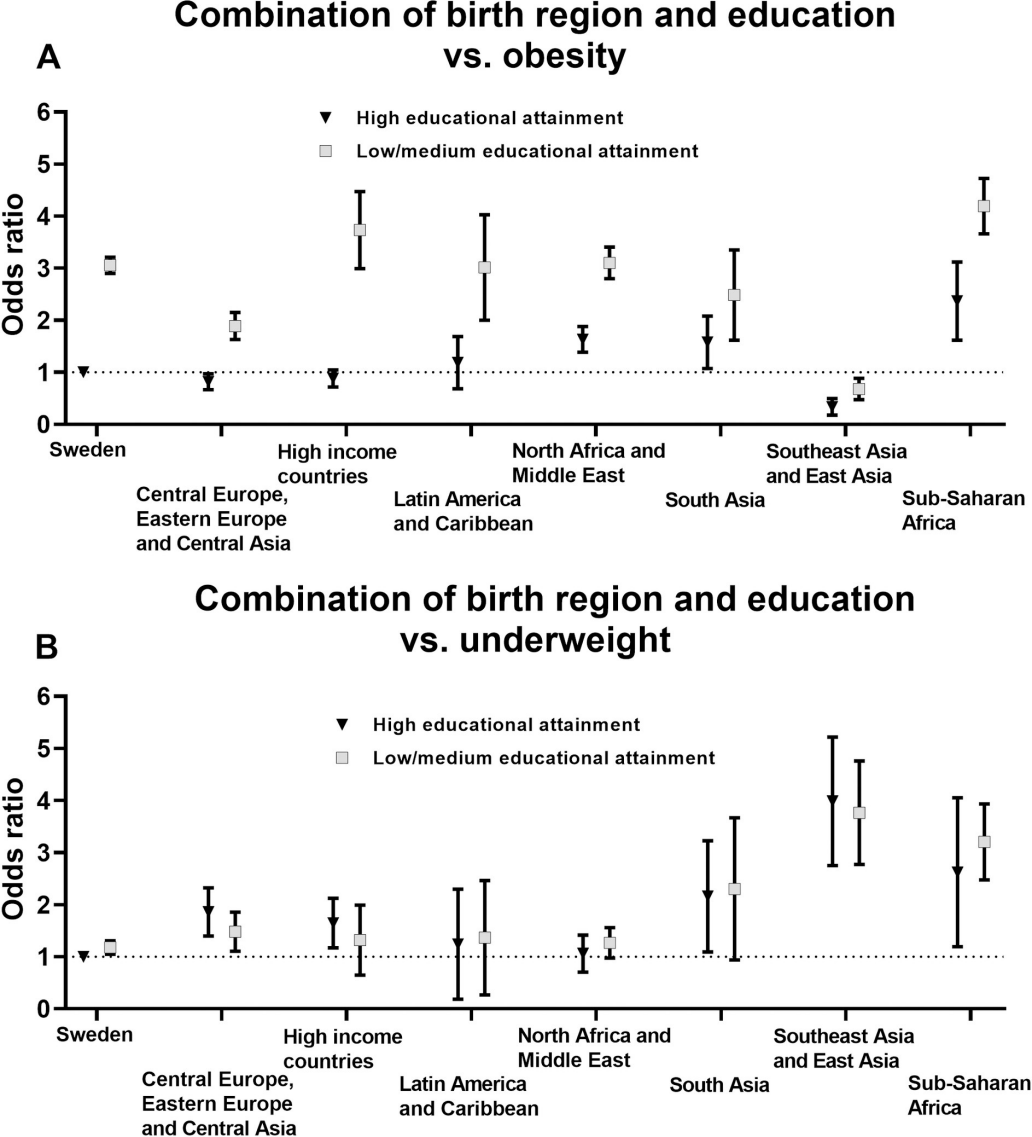
Odds ratios of (**A**) obesity and (**B**) underweight in the first trimester of pregnancy by combinations of birth regions and educational attainment. Multinomial logistic regression was used to estimate odds ratios with 95% confidence intervals (women born in Sweden with high educational attainment was reference group). Models were adjusted for age, parity and gestational age at first antenatal care visit.

**Fig 4:**
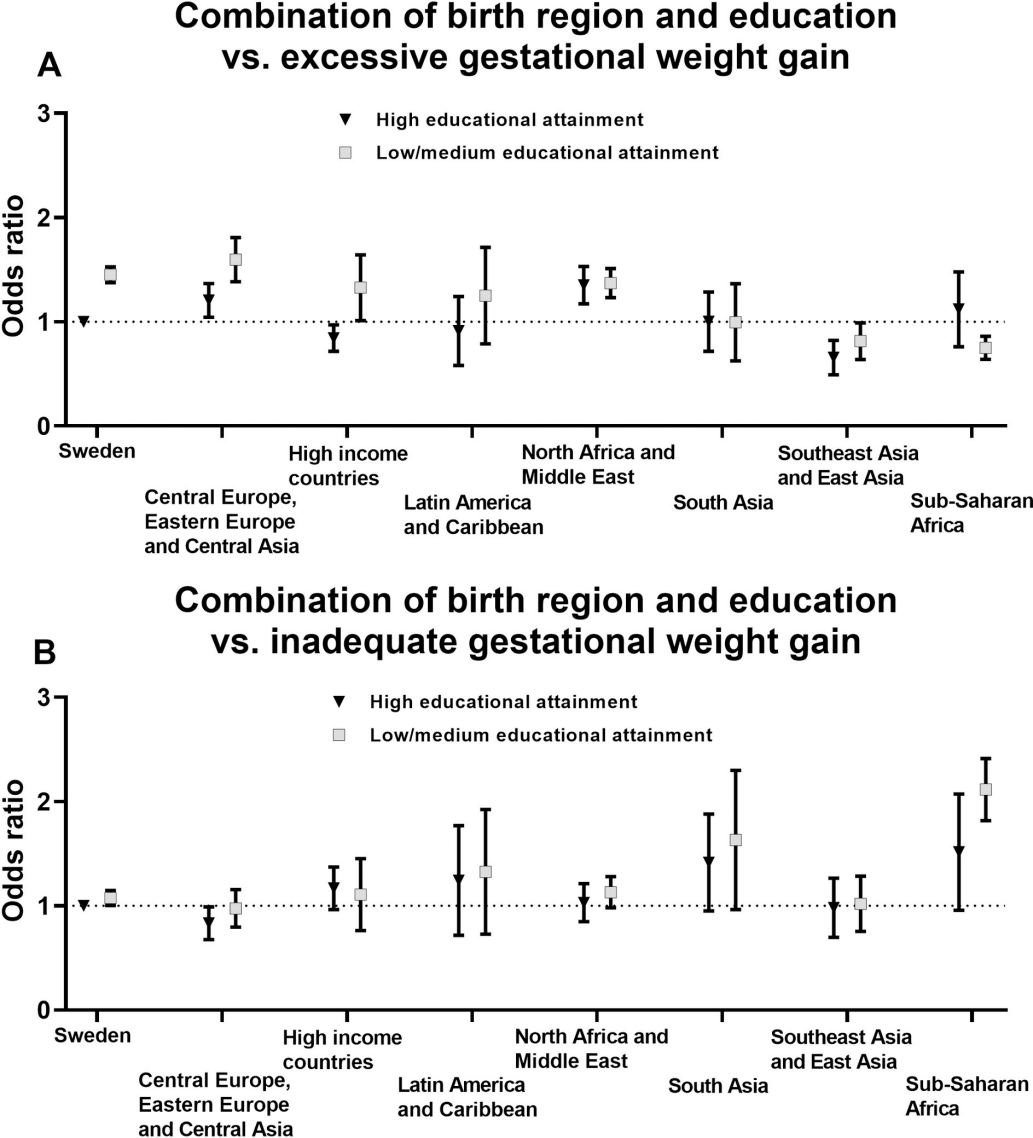
Odds ratios of (**A**) excessive and (**B**) inadequate gestational weight gain by combinations of birth regions and educational attainment. Multinomial logistic regression was used to estimate odds ratios with 95% confidence intervals (women born in Sweden with high educational attainment was reference group). Models were adjusted for age, parity and gestational age at first antenatal care visit.

ORs of unhealthy BMI and GWG by combinations of birth regions and educational attainment are presented in Fig 3 (obesity and underweight) and Fig 4 (excessive and inadequate GWG) and detailed data is found in S3 and S4 Tables. The results regarding the odds of obesity revealed a clear difference between low/middle and high educational attainment. Thus, as shown in Fig 3A, the odds of obesity were considerably greater for women with low/middle educational attainment as compared with women with high educational attainment in Sweden as well as in all seven super-regions. However, no similar pattern across levels of educational attainment were observed for the odds of underweight (Fig 3B).

Regarding GWG, women born in Sweden or other high-income countries with low/middle educational attainment had higher odds of excessive GWG as compared with peers with high educational attainment (Fig 4A). However, there were no large differences across birth regions in the odds of inadequate GWG between women with low/middle educational attainment as compared with women with high educational attainment (Fig 4B).

### Results for individual countries

Data for the 100 individual countries with the most registered pregnancies are presented in S5 Table. Generally, there were large differences in BMI and GWG across birth countries. Obesity was most prevalent in women born in Cameroon (30.1%), Democratic Republic of the Congo (28.8%), Chile (28.0%), Somalia (26.3%) and Egypt (25.6%) whereas the prevalence of underweight was highest in women born in Japan (11.5%), Kazakhstan (10.6%), China (10.6%), Eritrea (10.1%) and Vietnam (9.7%). Regarding GWG, the prevalence of excessive GWG was highest in women born in Montenegro (59.9%), Turkey (58.9%), Venezuela (58.0%), Bosnia and Herzegovina (57.9%) and Moldova (57.1%) whereas inadequate GWG was most common in women born in Japan (47.9%), Somalia (42.6%), The Gambia (36.1%), Yemen (34.1%) and Kenya (32.0%). There was also considerable heterogeneity in BMI and GWG between birth countries within regions. For instance, for eastern Sub-Saharan Africa the prevalence of obesity was considerably higher in women born in Somalia (26.3%) as compared with women born in Eritrea (7.3%) or Ethiopia (8.4%) whereas the risk of excessive GWG was lower for women born in Somalia (23.2% vs. 32.0% and 38.4%, respectively).

### Sensitivity analyses

We performed several sensitivity and additional analyses to examine the robustness of our findings. First, we examined the reasons for missing data in greater detail as shown in S6 Table. This analysis showed that the availability of BMI data was high across all birth regions (all ≥ 95.0%). However, there were relatively large differences in the availability of data regarding covariates and a registered body weight in the first trimester which led to the fact that Swedish-born women were more likely to be included in the analytic sample than women born outside Sweden. Therefore, we performed two sensitivity analyses which examined whether prevalence and unadjusted odds ratios across birth regions would be different if all available data (e.g. 95.2% of all women with a registered birth country in the BMI-analysis) were used as compared to the analytic sample. As shown in S7 and S8 Tables, the prevalence estimates and odds ratios remained very similar which corroborate the robustness of the reported prevalence and odds ratios of unhealthy BMI (underweight, obesity) and GWG (inadequate and excessive). However, we cannot exclude the possibility that the missing data may have had some influence on the adjusted estimates. Second, we classified GWG as inadequate, adequate, and excessive using the recommended weekly weight gains in the second and third trimester [3] and compared results to our main analysis (S9 Table). We also utilized the INTERGROWTH-21 weight gain standards [19] and compared the results with our main analysis for normal weight women (S10 Table). Although the prevalence of excessive and inadequate GWG differed somewhat between the calculations, the ORs for excessive and inadequate GWG across birth regions were very comparable to our main analysis. Third, we also examined differences in BMI and GWG across birth regions using quantile regression (10^th^, 50^th^ and 90^th^ percentile) as shown in S11 Table (BMI) and S12 Table (GWG). Finally, we performed an additional adjustment for the use of an interpreter in maternity care (yes vs. no) as a proxy for acculturation. As shown in S13 Table, this adjustment had very little influence on the estimates for obesity, underweight as well as excessive and inadequate GWG.

## Discussion

### Main findings

In this large Swedish population-based registry study, we found that women born in Sub-Saharan Africa or North Africa and Middle East had the greatest odds of obesity in the first trimester of pregnancy. Interestingly, women born in Sub-Saharan Africa were also at greater odds of underweight as compared with Swedish-born women. Furthermore, there was a clear pattern across all regions that women with low/middle educational attainment were considerably more likely to have obesity, which was not the case for underweight. Regarding GWG, women born in Central Europe, Eastern Europe and Central Asia had the highest odds of experiencing excessive GWG, in comparison to Swedish-born women, whereas women born in Sub-Saharan Africa had the lowest odds of excessive GWG. Conversely, women born in Sub-Saharan Africa were more likely to have inadequate GWG as compared with Swedish-born women. Importantly, it should be noted that there was considerable variation in BMI and GWG between the 100 examined birth countries, also within the same region.

### Interpretation of main findings

Our results are in line with previous European studies which have reported higher obesity prevalence in women born in or with origin in Sub-Saharan Africa [20–22], North Africa and Middle East [20, 22] or in specific countries/regions in Africa or Middle East such as Somalia [23], Lebanon [24], Morocco [25, 26] and Turkey [24–26]. The current study confirms, and also extends, these previous findings by providing obesity prevalence for all super-regions and 100 individual birth countries of which most are not covered by previous studies. There are fewer European studies that have examined birth country or origin in relation to underweight and these studies have shown somewhat conflicting results [21–24, 26]. For instance, Norwegian data [22] indicated that the prevalence of underweight was greater in women born in Sub-Saharan Africa although these results have not been observed in other studies [21, 23]. These conflicting results may be due to the fact that the prevalence of underweight in women born in Sub-Saharan Africa may vary by country. Indeed, although our results show that the odds of underweight in women born in Sub-Saharan Africa is around twice as compared with those born in Sweden, we observed a great variation in the prevalence of underweight across countries. Thus, our results highlight the importance of considering such country-specific differences in unhealthy BMI and GWG.

Only a few and relatively small studies have examined associations of birth regions/countries with excessive GWG within a European context, e.g. [25, 27–29]. Previous studies have indicated that women with non-European [28] and Moroccan [25, 27] origin may have lower risk for excessive GWG than non-migrant women, whereas Kinnunen et al. [29] did not identify any statistically significant differences across ethnic groups. Our findings show large variation in the odds of excessive GWG across birth regions although women born in Sweden had greater odds of excessive GWG than first-generation migrant women from some, but not all continents (i.e. Central Europe, Eastern Europe and Central Asia as well as North Africa and Middle East). Thus, our findings highlight the need of examining more detailed birth regions and countries in order to identify women at greater risk of excessive GWG. Furthermore, there is a paucity in the literature regarding the prevalence of inadequate GWG in migrant women. Our novel results demonstrate that inadequate GWG is common among pregnant first-generation migrant women, especially in women born in Sub-Saharan Africa (e.g. Somalia), which demonstrates the need to promote adequate GWG and not only the avoidance of excessive GWG.

### Strengths and limitations

This study has several distinct strengths including the population-based design utilizing a registry that covers approximately 90% of all contemporary births in Sweden. Furthermore, the large study size of more than 500 000 pregnancies enabled analysis of BMI and GWG for all super-regions as well as for 100 individual countries.

The study also has limitations that needs to be acknowledged. First, although we utilized body weight in the first trimester and the end of pregnancy to calculate a weight gain that covered the majority of the pregnancy (approximately 30 weeks of average), we did not have a pre-gestational weight as well as the weight at the time of the delivery. Second, this study had some missing data and women born in Sweden had generally more complete data of covariates and thus were more likely to be included in the analytic sample. Nevertheless, the proportion of available BMI data was equally high across all birth regions (all regions ≥ 95.0%) and our sensitivity analyses (S7 and S8 Tables) corroborated our prevalence data and unadjusted ORs. Third, although we observed distinct differences in BMI and GWG across birth regions, we cannot conclude exactly how much of these differences that are due to various covariates and mediating factors. Although we adjusted our estimates for potentially important covariates, we cannot exclude the possibility that the adjusted estimates would be influenced by covariates not accounted for in the current analysis. For instance, although our results were virtually unaffected by an interpreter in maternity care (as a crude proxy for acculturation), future studies should consider more accurate measures of acculturation. Furthermore, the use of self-reported educational attainment represents another limitation. Therefore, future studies could consider more comprehensive measures of covariates and mediators (e.g. accurate measures of acculturation, education, health conditions as well as health behaviors such as diet, physical activity, smoking and substance use) to further elucidate potential factors that underlie our observed differences in unhealthy BMI and GWG across birth regions. Fourth, the Swedish Pregnancy Register contains information on birth country but has no data regarding parental birth country and the reason for migration (e.g. labor or forced migration). Thus, future studies of BMI and GWG in migrant women should consider data on second-generation migration and reason for migration. Finally, our results may be useful in a European context, however, they may be less relevant for other regions and countries such as the USA with different migration patterns both in terms of sending and receiving countries as well as the reasons for migration [11].

### Public health and clinical implications

The findings of this study may have several implications for public health and clinical care. Our results provide prevalence of underweight, obesity as well as inadequate and excessive GWG in first-generation migrant women and demonstrate a large variation in pre-pregnancy BMI and GWG according to birth region/country. This information is essential since both BMI and GWG are modifiable factors that could be targeted to promote maternal and offspring health [2–5] and may be especially important for women at increased risk of adverse pregnancy outcomes [10, 11]. Furthermore, the categorization of women into birth regions has been hypothesized to mask differences across countries [30]. Indeed, our results demonstrate large heterogeneity in BMI and GWG between the 100 examined birth countries, also within the same region. For instance, BMI and GWG varied greatly between Eritrea, Ethiopia and Somalia despite being neighboring countries which highlights the need to also consider country-specific risks of unhealthy BMI and GWG in migrant women. These results also highlight the need for caution when comparing studies in the field considering that samples may contain various representation of birth countries which in turn may have very different risks of unhealthy BMI and GWG. Furthermore, our results also demonstrated the relevance of considering socioeconomic status in relation to BMI in early pregnancy, considering that the observed associations with obesity were greatly attenuated by adjustments for educational attainment. Altogether, our results may be of importance to identify groups of women at increased risk for both high and low BMI and GWG which is relevant for both public health strategies and clinical care. However, in order to tailor effective public health and clinical interventions, future studies are needed to further establish the causes, such as dietary and physical activity behaviors, environmental factors, social support etc. [8, 9], underlying the observed disparities in underweight, obesity and unhealthy GWG in this study.

## Conclusion

This Swedish population-based study of 0.5 million pregnancies demonstrates large disparities in unhealthy BMI and GWG across birth regions. For instance, women born in North Africa and Middle East and Sub-Saharan Africa had 1.4 and 2.1 greater odds of obesity in the first trimester of pregnancy as compared with women born in Sweden. However, women born in Sub-Saharan Africa also had around twice the odds of underweight and inadequate GWG which highlights the need of considering both excessive body weight and GWG as well as underweight and inadequate GWG in first-generation migrant pregnant populations. Thus, our findings also indicate that additional support and interventions may be needed for first-generation migrant women from certain birth regions and countries in order to tackle the observed disparities in unhealthy BMI (underweight and obesity) and GWG (inadequate and excessive). Although further studies are needed to clarify the underlying causes of the observed disparities in BMI and GWG, our results may be useful for identifying groups of women at increased risk of unhealthy BMI and GWG.

## Supporting information

S1 File RECORD checklist. (DOCX)

S2 File Prospective analysis plan. The original analysis plan sent to the Regional Ethical Review Board, Stockholm, Sweden (2018/656-31) in order to obtain ethical approval for the study.
(DOCX)

S3 File Supplementary methods. (DOCX)

S1 Table Odds ratios of obesity, overweight and underweight in the first trimester of pregnancy by birth regions. (DOCX)

S2 Table Odds ratios of excessive and inadequate Gestational Weight Gain (GWG) by birth regions. (DOCX)

S3 Table Odds ratios of obesity and underweight in the first trimester of pregnancy by combinations of birth regions and educational attainment. (DOCX)

S4 Table Odds ratios of excessive and inadequate gestational weight gain by combinations of birth regions and educational attainment. (DOCX)

S5 Table Body mass index in the first trimester of pregnancy and gestational weight gain according to individual birth country and regions. (DOCX)

S6 Table Availability of data in the database retrieved from the Swedish Pregnancy Register (2010–2018) stratified by birth region. (DOCX)

S7 Table Comparing the prevalence and unadjusted odds ratios of obesity and underweight by birth regions with all available data as compared to the analytic sample. (DOCX)

S8 Table Comparing the prevalence and unadjusted odds ratios of excessive and inadequate Gestational Weight Gain (GWG) by birth regions with all available data as compared to the analytic sample. (DOCX)

S9 Table Comparing the odds ratios of excessive and inadequate Gestational Weight Gain (GWG) by birth regions, using two GWG calculations. (DOCX)

S10 Table Comparing the odds ratios of excessive and inadequate Gestational Weight Gain (GWG) in normal weight women (n = 160 658) using the original calculation as compared to the cut-offs by INTERGROWTH-21. (DOCX)

S11 Table Differences in body mass index (kg/m^2^) in the first trimester of pregnancy by birth regions as calculated by means of quantile regression. (DOCX)

S12 Table Differences in gestational weigh gain (kg per week) by birth regions as calculated by means of quantile regression. (DOCX)

S13 Table Comparing the odds ratios of obesity, underweight as well as excessive and inadequate Gestational Weight Gain (GWG) by birth regions, with additional adjustments for the use of an interpreter in maternity care (yes vs. no) as a proxy for acculturation. (DOCX)
