# Supplementary material for: Body mass index and gestational weight gain in migrant women by birth regions compared with Swedish-born women: A registry linkage study of 0.5 million pregnancies
Source: PLoS One. 2020 Oct 29;15(10):e0241319. doi: 10.1371/journal.pone.0241319 (PMC7595374; doi:10.1371/journal.pone.0241319)
Supplement: S2 File — The original analysis plan sent to the Regional Ethical Review Board, Stockholm, Sweden (2018/656-31) in order to obtain ethical approval for the study. (DOCX) [file pone.0241319.s002.docx]

**Associations of migrant and socioeconomic status with BMI,**

**gestational weight gain and self-reported health:**

**a population-based study from the Swedish Pregnancy Registry**

**Background**

Excessive body weight and gestational weight gain are important modifiable risk factors for numerous pregnancy outcomes for both mother and child (1). For instance, both overweight/obesity before or in early pregnancy and excessive gestational weight gain has been associated with increased risks for adverse pregnancy health outcomes such as gestational diabetes, preeclampsia, cesarean delivery and infant death (1-4). Conversely, underweight and inadequate gestational weight gain have been associated with higher risks of low birth weight infants and preterm birth (1).

It is well-established that women being born in a foreign country, herein referred to as “migrant women”, have higher risks for severe maternal morbidity as well as maternal and infant death compared to women born in Sweden (5-7). However, it is not known whether the prevalence of unhealthy weight status, GWG and poor self-reported health is different among migrant women or women born in Sweden. It may for instance be hypothesized that for some migrant women the prevalence of unhealthy weight status, GWG and poor self-reported health is more common than among women born in Sweden. However, no such Swedish data is currently available. Furthermore, the few such studies have been conducted globally (for instance; 8-12) have presented mixed findings and majority have been relatively small (9-12). Furthermore, the association of socioeconomic status with weight status, gestational weight gain and self-reported health is relatively unknown, especially in migrant women. Gaining more knowledge in this area is critical since migrant health is a great public health priority and challenge (13, 14) and many countries including Sweden have a steadily increasing migrant population in childbearing age. Indeed, 27 % of women giving birth in 2016 in Sweden were born in another country (15).

The study described in the following application is funded by Forte (dnr: 2017-00088, principal investigator: Pontus Henriksson) and will increase the knowledge regarding the associations of migrant and socioeconomic status with BMI, gestational weight gain and health in a large (> 500 000) population-based cohort of pregnant women with various migrant and socioeconomic backgrounds.

**Specific aims**

The specific aims of this study are to examine associations of i) migrant status ii) socioeconomic status and iii) the combination of migrant and socioeconomic status with

1. BMI and weight status in early pregnancy
2. Gestational weight gain
3. Self-reported health before, during and after pregnancy

**Hypothesis**

We hypothesize that women with migrant background and/or low socioeconomic status have higher pre-pregnancy BMI and gestational weight gain and lower self-reported health before, during and after pregnancy.

**Project description**

**Study design**

Data of exposures, outcomes and covariates (see below) will be retrieved from the Swedish Pregnancy Register for the years 2010 to 2018 and study will include approximately 500 000 women. An internal validation of this registry showed that variables within this registry has overall good to very good degree of coverage, agreement and internal validity (16).

***Main exposure variables***

- Country of birth
- Need of an interpreter in contact with maternity care
- Educational attainment (corresponding to < 9 years, 9-12 years, > 12 years)
- Occupation (work, student, parental leave, unemployed, sick leave/disability pension, others)

***Main outcomes variables***

- BMI, weight and height in early and late pregnancy (kg/m^2^)
- Weight status categories in early pregnancy
- Gestational weight gain between early (at first visit at maternity care) and late pregnancy (after gestational week 35) (kg)
- Gestational weight gain according to IOM recommendations (1)
- Gestational weight gain z-scores (17)
- Self-reported health before, during and after pregnancy

***Covariates and descriptive variables***

Following variables will be considered as confounders in the analysis and will also be utilized as descriptive data; maternal age, number of previous child labors, previous pregnancies in the Swedish Pregnancy Registry, number of born children in current pregnancy (i.e. single or multiple-pregnancy), length of pregnancy, county council, maternity care unit, smoking, alcohol consumption, type of delivery, infant sex, birth weight and date, stillbirth, dates for visits in maternity care and revisits after birth, educational attainment and occupation, estimated day of birth from ultrasound and last menstruation.

***Data analysis and power calculations***

Differences between groups will be analyzed by means of independent t-tests and Analysis of Covariance (continuous outcomes) or logistic regression (categorical outcomes). The power of the study is high. For instance, with a sample of 500 000 women we will be able to detect a 0.015 standard deviations (SDs) difference in BMI, gestational weight gain and self-reported health between migrant and non-migrant women with a power of 95 % (α=0.05). This corresponds to differences of 0.07 kg/m^2^ (assumed SD = 4.8), 0.06 kg (assumed SD = 4 kg) and 0.005 points (assumed SD = 0.3 points) in BMI, gestational weight gain and self-reported health respectively (15, 18). Given the large sample size we will also analyze GWG according to region of birth (6), economic status (low/middle/high) of birth country (7) and the combination between socioeconomic and migrant status.

**Previous experiences of methods/procedures and safety for participants**

The research group consists of Pontus Henriksson (Registered Dietitian and PhD), Marie Löf (Associate Professor), Marie Blomberg (Senior Consultant Physician and Associate Professor), Kerstin Petersson (Midwife and PhD) and Paulina Nowicka (Associated Professor and Registered Dietitian). Altogether, the research group has extensive experiences of conducting large scale epidemiological studies and registry studies, for instance (16, 19-25)

**Ethical considerations and safety of participants**

Since the data in this study is collected retrospectively and anonymously, there are minimal risks with the study. The study may aid in promoting health of pregnant women with various socioeconomic and migrant backgrounds. Hence, we believe this study is ethically justified.

**Significance**

Migrant health represents a great public health priority and challenge (13, 14) and currently approximately 27 % of pregnant women in Sweden have migrant background (15). Pre-pregnancy body mass index and GWG is a modifiable risk factor for a wide range of pregnancy health outcomes including mortality and severe morbidity in both mother and child (1-4). Hence, this large population based registry study (n > 500 000) will provide novel data of weight status in early pregnancy, gestational weight gain and self-reported health in women with various socioeconomic and migrant background. A distinct study strength is the use of the Swedish Pregnancy Register which has is population-based and has good coverage (16). Furthermore, the large sample size will also allow us to provide data for GWG of migrant women stratified by geographical regions (6), economic status of birth country (7), as well as to examine whether inequalities in GWG can be attenuated by higher educational attainment or occupational status. All of this information is essential, even though the previous literature has demonstrated that migrant women have an increased risk for severe adverse pregnancy outcomes (5-7), less is known whether migrant women have an increased risk of unhealthy weight status, GWG and poor self-reported health.

Altogether, this project will provide evidence whether inequalities regarding weight status, gestational weight gain and self-reported health exist between women with migrant and non-migrant women and/or with low and high socioeconomic status. The data in this described project will therefore contribute to the existing literature. Furthermore, the study has also a great potential to aid in the development of appropriate clinical care and public health polices to promote the health of pregnant women with various socioeconomic and migrant backgrounds.

**References**

1. Institute of Medicine. Weight gain during pregnancy: reexamining the guidelines, 2009.

2. Bodnar LM et al. Maternal obesity and gestational weight gain are risk factors for infant death. Obesity 2016;24:490-8.

3. Brunner S et al. Excessive gestational weight gain prior to glucose screening and the risk of gestational diabetes: a meta-analysis. Diabetologia 2015;58:2229-37.

4. Ferraro ZM et al. Obstet Med. 2015;8:133-7.

5. Gagnon AJ et al. Migration to western industrialised countries and perinatal health: a systematic review. Soc Sci Med 2009;69:934-46.

6. Urquia ML et al. Severe maternal morbidity associated with maternal birthplace in three high-immigration settings. Eur J Public Health 2015;25:620-5.

7. Wahlberg A et al. Increased risk of severe maternal morbidity (near-miss) among immigrant women in Sweden: a population register-based study. BJOG 2013;120:1605-11.

8. Deputy NP et al. Prevalence and characteristics associated with gestational weight gain adequacy. Obstet Gynecol 2015;125:773-81.

9. Restall A et al. Risk factors for excessive gestational weight gain in a healthy, nulliparous cohort. J Obes 2014;2014:148391.

10. Bastola K et al. Pre-pregnancy body mass index and inter-pregnancy weight change among women of Russian, Somali and Kurdish origin and the general Finnish population. Scand J Public Health. 2017;45:314-21.

11. Larouche M et al. The effect of recent immigration to Canada on gestational weight gain. J Obstet Gynaecol Can. 2010;32:829-36.

12. Alder J et al. The effect of migration background on obstetric performance in Switzerland. Eur J Contracept Reprod Health Care. 2008;13:103-8.

13. Jakab et al. Refugee and migrant health. Lancet 2015,386:2477-8.

14. The National Board of Health and Welfare; http://www.socialstyrelsen.se/publikationer2016/2016-10-13

15. Swedish Pregnancy Register: graviditetsregistret.se.

16. Petersson K et al. Internal validity of the Swedish Maternal Health Care Register. BMC Health Serv Res 2014;14:364.

17. Johansson K et al. Pregnancy weight gain by gestational age and BMI in Sweden: a population-based cohort study. Am J Clin Nutr 2016;103:1278-84.

18. Ruiz JR et al. Supervised exercise-based intervention to prevent excessive gestational weight gain: a randomized controlled trial. Mayo C lin Proc 2013;88:1388-97.

19. Henriksson P et al. Prevalence of ideal cardiovascular health in European adolescents: The HELENA Study. Int J Cardiol. 2017;240:428-432.

20. Henriksson P et al. Correlates of ideal cardiovascular health in European adolescents: The HELENA Study. Accepted in Nutr Metab Cardiovasc Dis.

21. Löf M et al. Prospective study of coffee consumption and all-cause, cancer, and cardiovascular mortality in Swedish women. Eur J Epidemiol 2015;30: 1027-34.

22. Löf M et al. Fruit and vegetable intake and risk of cancer in the Swedish women's lifestyle and health cohort. Cancer causes & control 2011; 22:283-9.

23. Blomberg M et al. Impact of maternal age on obstetric and neonatal outcome with emphasis on primiparous adolescents and older women: a Swedish Medical Birth Register Study. BMJ open 2014; 4(11):e005840

24. Blomberg M et al. Maternal body mass index and risk of obstetric anal sphincter injury. BioMed research international 2014;2014:395803.

25. Nowicka P et al. Assessment of parental overt and covert control of child's food intake: a population-based validation study with mothers of preschoolers. Eating behaviors 2014;15: 673-8.
