# Supplementary material for: Body mass index and gestational weight gain in migrant women by birth regions compared with Swedish-born women: A registry linkage study of 0.5 million pregnancies
Source: PLoS One. 2020 Oct 29;15(10):e0241319. doi: 10.1371/journal.pone.0241319 (PMC7595374; doi:10.1371/journal.pone.0241319)
Supplement: S3 File — (DOCX) [file pone.0241319.s003.docx]

**S3 File. Supplementary methods.** Additional methods regarding the calculations and categorization of gestational weight gain as inadequate, adequate, and excessive according to

National Academy of Medicine.

GWG was calculated as body weight in late pregnancy minus body weight at the first antenatal visit. We then utilized the GWG recommendations by the National Academy of Medicine (REF), USA, (formerly Institute of Medicine) (i.e. underweight: 12.5-18.0 kg; normal weight: 11.5-16.0 kg; overweight: 7.0-11.5 kg; obesity: 5.0-9.0 kg). To account for the fact that women attend antenatal visits before labor, we individually tailored these GWG cut-offs for the recommended weekly gains in the second and third trimester [3]. (i.e. underweight: 0.51 kg/week; normal weight: 0.42 kg/week; overweight: 0.28 kg/week; obesity: 0.22 kg/week) so they would be appropriate for the gestational age in which their last body weight was measured.

For instance, for a normal weight woman that had her last weight measured at gestational week 38+6 the recommended GWG would be 11.08-15.58 (i.e. 11.5 and 16.0 minus 0.42). Corresponding figure for a normal weight woman with a final measurement at 40+5 would be 11.86-16.36 (i.e. 11.5 and 16.0 plus 0.36 [0.42 x 6/7]), i.e. 6/7 of a week should be added to the recommendation in this case.

**Example of categorization.**

|  | **Gestational weight gain recommendation**  **according gestational age at final weight** | | | | | |
| --- | --- | --- | --- | --- | --- | --- |
| **BMI-categories** | 36+6^1^ | 37+6 | 38+6 | 39+6^2^ | 40+6 | 41+6 |
| Underweight | 10.97-  16.47 | 11.48-  16.98 | 11.99-  17.49 | 12.5-  18.0 | 13.01-  18.51 | 13.51-  19.02 |
| Normal weight | 10.24-  14.74 | 10.66-  15.16 | 11.08-  15.58 | 11.5-  16.0 | 11.92-  16.42 | 12.34-  16.84 |
| Overweight | 6.16-  10.66 | 6.44-  10.94 | 6.72-  11.22 | 7.0-  11.5 | 7.28-  11.78 | 7.56-  12.06 |
| Obesity | 4.34-  8.34 | 4.56-  8.56 | 4.78-  8.78 | 5.0-  9.0 | 5.22-  9.22 | 5.44-  9.44 |

^1^ Please note that tailoring was made on the exact gestational age and not rounded to weeks although the table only provides data for exact weeks below or above date of estimated birth.

**^2^** National Academy of Medicine recommendation for GWG for an entire pregnancy.
