# Supplementary material for: Body mass index and gestational weight gain in migrant women by birth regions compared with Swedish-born women: A registry linkage study of 0.5 million pregnancies
Source: PLoS One. 2020 Oct 29;15(10):e0241319. doi: 10.1371/journal.pone.0241319 (PMC7595374; doi:10.1371/journal.pone.0241319)
Supplement: S1 Table — (DOCX) [file pone.0241319.s004.docx]

**S1 Table.** Odds ratios of obesity, overweight and underweight in the first trimester of pregnancy by birth regions^1^.

| **Obesity** | **Prevalence** | **OR (95 % CI)** | | **OR (95 % CI)** | **OR (95 % CI)** |
| --- | --- | --- | --- | --- | --- |
| **Birth region** |  | ***Unadjusted*** | | ***Basic adjustment^2^*** | ***Basic adjustment^2^ + education*** |
| Sweden | 12.7 % | Reference | | Reference | Reference |
| Central Europe, Eastern Europe and Central Asia | 10.1 % | 0.76 (0.73-0.80)*** | | 0.75 (0.72-0.79)*** | 0.66 (0.64-0.70)*** |
| High income countries | 11.9 % | 0.90 (0.86-0.95)*** | | 0.92 (0.87-0.97)** | 0.99 (0.94-1.05) |
| Latin America and Caribbean | 13.5 % | 1.17 (1.05-1.30)** | | 1.18 (1.06-1.31) ** | 1.04 (0.93-1.15) |
| North Africa and Middle East | 15.3 % | 1.47 (1.42-1.51)*** | | 1.40 (1.35-1.44)*** | 1.08 (1.04-1.12)*** |
| South Asia | 12.3 % | 1.08 (0.98-1.19) | | 1.08 (0.98-1.18) | 1.10 (1.00-1.21) |
| Southeast Asia and East Asia | 4.6 % | 0.32 (0.29-0.35)*** | | 0.32 (0.29-0.35)*** | 0.23 (0.21-0.26)*** |
| Sub-Saharan Africa | 20.0 % | 2.21 (2.11-2.31)*** | | 2.13 (2.03-2.23)*** | 1.32 (1.25-1.38)*** |
|  |  | |  | | |
| **Overweight** | **Prevalence** | **OR (95 % CI)** | | **OR (95 % CI)** | **OR (95 % CI)** |
| **Birth region** |  | ***Unadjusted*** | | ***Basic adjustment^2^*** | ***Basic adjustment^2^ + education*** |
| Sweden | 24.4 % | Reference | | Reference | Reference |
| Central Europe, Eastern Europe and Central Asia | 22.9 % | 0.90 (0.87-0.93)*** | | 0.89 (0.87-0.92)*** | 0.85 (0.82-0.88)*** |
| High income countries | 22.4 % | 0.88 (0.85-0.92)*** | | 0.88 (0.84-0.92)*** | 0.91 (0.88-0.95)*** |
| Latin America and Caribbean | 29.2 % | 1.31 (1.22-1.42)*** | | 1.31 (1.21-1.41)*** | 1.23 (1.14-1.34)*** |
| North Africa and Middle East | 32.6 % | 1.63 (1.59-1.67)*** | | 1.59 (1.55-1.63)*** | 1.43 (1.39-1.47)*** |
| South Asia | 29.1 % | 1.33 (1.24-1.43)*** | | 1.33 (1.24-1.43)*** | 1.36 (1.27-1.46)*** |
| Southeast Asia and East Asia | 17.1 % | 0.61 (0.58-0.65)*** | | 0.61 (0.57-0.64)*** | 0.53 (0.50-0.56)*** |
| Sub-Saharan Africa | 31.7 % | 1.82 (1.75-1.90)*** | | 1.78 (1.71-1.85)*** | 1.43 (1.38-1.50)*** |
|  |  | |  | | |
| **Underweight** | **Prevalence** | **OR (95 % CI)** | | **OR (95 % CI)** | **OR (95 % CI)** |
| **Birth region** |  | ***Unadjusted*** | | ***Basic adjustment^2^*** | ***Basic adjustment^2^ + education*** |
| Sweden | 2.3 % | Reference | | Reference | Reference |
| Central Europe, Eastern Europe and Central Asia | 3.6 % | 1.55 (1.44-1.66)*** | | 1.54 (1.43-1.65)*** | 1.47 (1.37-1.58)*** |
| High income countries | 2.9 % | 1.23 (1.11-1.35)*** | | 1.44 (1.30-1.59)*** | 1.43 (1.30-1.59)*** |
| Latin America and Caribbean | 2.2 % | 1.06 (0.83-1.34) | | 1.18 (0.93-1.49) | 1.14 (0.90-1.45) |
| North Africa and Middle East | 2.2 % | 1.19 (1.11-1.29)*** | | 1.12 (1.04-1.21)** | 1.00 (0.93-1.09) |
| South Asia | 4.1 % | 2.04 (1.75-2.37)*** | | 2.05 (1.76-2.39)*** | 2.00 (1.71-2.33)*** |
| Southeast Asia and East Asia | 8.3 % | 3.20 (2.95-3.47)*** | | 3.61 (3.33-3.91)*** | 3.27 (3.01-3.55)*** |
| Sub-Saharan Africa | 5.0 % | 3.07 (2.83-3.33)*** | | 2.93 (2.70-3.18)*** | 2.40 (2.20-2.62)*** |

**P* < 0.05, ***P* < 0.01, ****P* < 0.001.

^1^ Calculated by means of multinomial logistic regression (normal weight = reference).

^2^ Basic adjustments in the analyses were age, parity and gestational age at first antenatal care visit.
