# Supplementary material for: Body mass index and gestational weight gain in migrant women by birth regions compared with Swedish-born women: A registry linkage study of 0.5 million pregnancies
Source: PLoS One. 2020 Oct 29;15(10):e0241319. doi: 10.1371/journal.pone.0241319 (PMC7595374; doi:10.1371/journal.pone.0241319)
Supplement: S2 Table — (DOCX) [file pone.0241319.s005.docx]

**S2 Table.** Odds ratios of excessive and inadequate gestational weight gain (GWG) by birth regions^1^.

| **Excessive GWG** | **Prevalence** | **OR (95 % CI)** | | **OR (95 % CI)** | **OR (95 % CI)** |
| --- | --- | --- | --- | --- | --- |
| **Birth region** |  | ***Unadjusted*** | | ***Basic adjustment^2^*** | ***Basic adjustment^2^ + education*** |
| Sweden | 47.4 % | Reference | | Reference | Reference |
| Central Europe, Eastern Europe and Central Asia | 52.4 % | 1.17 (1.13-1.22)*** | | 1.18 (1.14-1.23)*** | 1.16 (1.12-1.21)*** |
| High income countries | 41.0 % | 0.80 (0.76-0.84)*** | | 0.84 (0.79-0.88)*** | 0.86 (0.82-0.91)*** |
| Latin America and Caribbean | 42.5 % | 0.89 (0.80-0.98)* | | 0.92 (0.83-1.02) | 0.89 (0.80-0.99)* |
| North Africa and Middle East | 50.1 % | 1.14 (1.11-1.18)*** | | 1.15 (1.11-1.18)*** | 1.11 (1.08-1.15)*** |
| South Asia | 40.2 % | 0.85 (0.78-0.94)** | | 0.84 (0.77-0.92)*** | 0.87 (0.79-0.95)** |
| Southeast Asia and East Asia | 36.2 % | 0.62 (0.58-0.67)*** | | 0.64 (0.60-0.68)*** | 0.62 (0.58-0.66)*** |
| Sub-Saharan Africa | 30.4 % | 0.65 (0.61-0.68)*** | | 0.67 (0.64-0.71)*** | 0.62 (0.59-0.66)*** |
|  |  | |  | | |
| **Inadequate GWG** | **Prevalence** | **OR (95 % CI)** | | **OR (95 % CI)** | **OR (95 % CI)** |
| **Birth region** |  | ***Unadjusted*** | | ***Basic adjustment^2^*** | ***Basic adjustment^2^ + education*** |
| Sweden | 17.5 % | Reference | | Reference | Reference |
| Central Europe, Eastern Europe and Central Asia | 14.5 % | 0.88 (0.83-0.93)*** | | 0.87 (0.83-0.92)*** | 0.85 (0.81-0.90)*** |
| High income countries | 21.1 % | 1.12 (1.05-1.19)** | | 1.13 (1.06-1.20)*** | 1.12 (1.06-1.20)*** |
| Latin America and Caribbean | 21.9 % | 1.24 (1.09-1.40)** | | 1.24 (1.09-1.40)** | 1.21 (1.07-1.37)** |
| North Africa and Middle East | 17.4 % | 1.07 (1.03-1.12)** | | 1.06 (1.02-1.11)** | 0.99 (0.95-1.04) |
| South Asia | 24.9 % | 1.44 (1.30-1.59)*** | | 1.44 (1.30-1.60)*** | 1.42 (1.28-1.58)*** |
| Southeast Asia and East Asia | 20.8 % | 0.97 (0.90-1.05) | | 0.97 (0.90-1.05) | 0.92 (0.85-1.00)* |
| Sub-Saharan Africa | 34.7 % | 2.00 (1.90-2.11)*** | | 1.97 (1.87-2.07)*** | 1.73 (1.63-1.83)*** |

**P* < 0.05, ***P* < 0.01, ****P* < 0.001.

^1^ Calculated by means of multinomial logistic regression (adequate GWG = reference).

^2^ Basic adjustments in the analyses were age, parity and gestational age at first antenatal care visit.
