# Supplementary material for: Body mass index and gestational weight gain in migrant women by birth regions compared with Swedish-born women: A registry linkage study of 0.5 million pregnancies
Source: PLoS One. 2020 Oct 29;15(10):e0241319. doi: 10.1371/journal.pone.0241319 (PMC7595374; doi:10.1371/journal.pone.0241319)
Supplement: S4 Table — (DOCX) [file pone.0241319.s007.docx]

**S4 Table.** Odds ratios of excessive and inadequate gestational weight gain by combinations of birth regions and educational attainment. Models were adjusted for age, parity and gestational age at first antenatal care visit^1^.

|  |  | **Excessive gestational weight gain** | |  |  | **Inadequate gestational weight gain** | |
| --- | --- | --- | --- | --- | --- | --- | --- |
| **Birth region** |  | **Prevalence** | **OR (95 % CI)** |  |  | **Prevalence** | **OR (95 % CI)** |
| **Sweden** |  |  |  |  | |  |  |
| High educational attainment |  | 43.3 % | Reference |  | | 18.5 % | Reference |
| Low/middle educational attainment |  | 52.7 % | 1.45 (1.42-1.48)*** |  | | 16.1 % | 1.08 (1.05-1.10)*** |
| **Central Europe, Eastern Europe and Central Asia** |  |  |  |  | |  |  |
| High educational attainment |  | 49.4 % | 1.21 (1.14-1.27)*** |  | | 14.5 % | 0.83 (0.77-0.89)*** |
| Low/middle educational attainment |  | 55.0 % | 1.60 (1.51-1.68)*** |  | | 14.5 % | 0.97 (0.90-1.05) |
| **High income countries** |  |  |  |  | |  |  |
| High educational attainment |  | 37.8 % | 0.84 (0.79-0.90)*** |  | | 22.4 % | 1.17 (1.09-1.25)*** |
| Low/middle educational attainment |  | 49.4 % | 1.32 (1.20-1.46)*** |  | | 17.7 % | 1.10 (0.97-1.25) |
| **Latin America and Caribbean** |  |  |  |  | |  |  |
| High educational attainment |  | 39.2 % | 0.91 (0.78-1.05) |  | | 22.6 % | 1.23 (1.04-1.46)* |
| Low/middle educational attainment |  | 46.0 % | 1.24 (1.07-1.44)** |  | | 21.1 % | 1.31 (1.09-1.57)** |
| **North Africa and Middle East** |  |  |  |  | |  |  |
| High educational attainment |  | 50.7 % | 1.35 (1.28-1.43)*** |  | | 16.4 % | 1.03 (0.96-1.10) |
| Low/middle educational attainment |  | 49.8 % | 1.37 (1.32-1.43)*** |  | | 17.9 % | 1.13 (1.07-1.19)*** |
| **South Asia** |  |  |  |  | |  |  |
| High educational attainment |  | 41.1 % | 1.00 (0.89-1.12) |  | | 23.7 % | 1.41 (1.23-1.61)*** |
| Low/middle educational attainment |  | 38.6 % | 0.99 (0.85-1.15) |  | | 27.0 % | 1.62 (1.37-1.91)*** |
| **Southeast Asia and East Asia** |  |  |  |  | |  |  |
| High educational attainment |  | 34.0 % | 0.66 (0.59-0.72)*** |  | | 21.0 % | 0.98 (0.87-1.10) |
| Low/middle educational attainment |  | 37.9 % | 0.81 (0.74-0.89)*** |  | | 20.5 % | 1.02 (0.91-1.13) |
| **Sub-Saharan Africa** |  |  |  |  | |  |  |
| High educational attainment |  | 41.9 % | 1.11 (0.98-1.27) |  | | 24.5 % | 1.50 (1.30-1.74)*** |
| Low/middle educational attainment |  | 28.5 % | 0.75 (0.71-0.80)*** |  | | 36.4 % | 2.11 (2.00-2.24)*** |

**P* < 0.05, ***P* < 0.01, ****P* < 0.001.

^1^ Calculated by means of multinomial logistic regression (adequate GWG = reference).
