# Supplementary material for: Body mass index and gestational weight gain in migrant women by birth regions compared with Swedish-born women: A registry linkage study of 0.5 million pregnancies
Source: PLoS One. 2020 Oct 29;15(10):e0241319. doi: 10.1371/journal.pone.0241319 (PMC7595374; doi:10.1371/journal.pone.0241319)
Supplement: S5 Table — (DOCX) [file pone.0241319.s008.docx]

**S5 Table.** Body mass index in the first trimester of pregnancy and gestational weight gain according to individual birth country and regions.

|  |  | **Body mass index (BMI) early in pregnancy** | | | | | |  | **Gestational weight gain (GWG)**^1^ | | | | |
| --- | --- | --- | --- | --- | --- | --- | --- | --- | --- | --- | --- | --- | --- |
| **Birth region/county** | | **n** | **BMI (kg/m^2^)** | **Under-weight**  **(%)** | **Normalweight**  **(%)** | **Over-weight**  **(%)** | **Obesity**  **(%)** |  | **n** | **GWG^2^**  **(kg)** | **Inadequate GWG**  **(%)** | **Adequate**  **GWG (%)** | **Excessive GWG**  **(%)** |
| **All countries** | | 535 609 | 24.7 ± 4.7 | 2.5 % | 59.7 % | 25.0 % | 12.8 % |  | 270 044 | 13.5 ± 5.1 | 18.1 % | 35.0 % | 46.8 % |
| **Sweden** | | 431 978 | 24.7 ± 4.7 | 2.3 % | 60.6 % | 24.4 % | 12.7 % |  | 209 454 | 13.6 ± 5.1 | 17.5 % | 35.1 % | 47.4 % |
| **Central Europe, Eastern Europe and Central Asia** | | 24 045 | 24.1 ± 4.4 | 3.6 % | 63.3 % | 22.9 % | 10.1 % |  | 14 277 | 14.4 ± 5.3 | 14.5 % | 33.2 % | 52.4 % |
| ***Central Asia*** | | 1 739 | 23.8 ± 4.2 | 5.0 % | 62.4 % | 24.0 % | 8.5 % |  | 1 098 | 13.7 ± 4.8 | 16.1 % | 36.3 % | 47.5 % |
| Armenia | | 306 | 24.1 ± 4.3 | 2.6 % | 64.4 % | 21.9 % | 11.1 % |  | 174 | 13.9 ± 5.1 | 17.2 % | 33.9 % | 48.9 % |
| Azerbaijan | | 227 | 24.6 ± 4.3 | 4.0 % | 55.9 % | 30.0 % | 10.1% |  | 143 | 13.6 ± 4.7 | 13.3 % | 30.8 % | 55.9 % |
| Georgia | | 116 | 23.5 ± 4.3 | 8.6 % | 57.8 % | 25.9 % | 7.8 % |  | 64 | 15.1 ± 5.0 | 7.8 % | 35.9 % | 56.3 % |
| Kazakhstan | | 189 | 22.4 ± 3.6 | 10.6 % | 65.6 % | 20.1 % | 3.7 % |  | 115 | 13.8 ± 4.2 | 19.1 % | 39.1 % | 41.7 % |
| Kyrgyzstan | | 100 | 23.1 ± 3.9 | 8.0 % | 64.0 % | 23.0 % | 5.0 % |  | 66 | 13.0 ± 4.0 | 21.2 % | 37.9 % | 40.9 % |
| Mongolia | | 394 | 23.7 ± 3.9 | 2.8 % | 67.3 % | 23.1 % | 6.9 % |  | 275 | 14.0 ± 4.7 | 14.2 % | 37.8 % | 48.0 % |
| Uzbekistan | | 351 | 23.9 ± 4.6 | 5.4 % | 61.3 % | 22.5 % | 10.8 % |  | 227 | 13.3 ± 5.2 | 18.5 % | 38.8 % | 42.7 % |
| ***Central Europe*** | | 17 376 | 24.4 ± 4.4 | 3.2 % | 61.7 % | 24.0 % | 11.2 % |  | 10 216 | 14.5 ± 5.4 | 14.0 % | 31.9 % | 54.0 % |
| Albania | | 1 447 | 24.4 ± 4.3 | 3.7 % | 60.3 % | 24.7 % | 11.3 % |  | 945 | 14.4 ± 5.5 | 14.6 % | 30.3 % | 55.1 % |
| Bosnia and  Herzegovina | | 4 398 | 24.5 ± 4.3 | 2.3 % | 61.3 % | 25.1 % | 11.4 % |  | 2 389 | 15.1 ± 5.5 | 11.3 % | 30.8 % | 57.9 % |
| Bulgaria | | 527 | 24.4 ± 4.6 | 3.6 % | 60.9 % | 23.3 % | 12.1 % |  | 314 | 14.1 ± 5.0 | 15.6 % | 34.1 % | 50.3 % |
| Croatia | | 746 | 24.1 ± 4.3 | 3.4 % | 64.3 % | 24.1 % | 8.2 % |  | 422 | 15.1 ± 5.6 | 13.3 % | 31.8 % | 55.0 % |
| Czech Republic | | 202 | 23.1 ± 3.9 | 3.0 % | 76.2 % | 12.4 % | 8.4 % |  | 117 | 13.1 ± 4.2 | 20.5 % | 41.0 % | 38.5 % |
| Hungary | | 567 | 24.1 ± 4.5 | 4.6 % | 62.6 % | 21.3 % | 11.5 % |  | 343 | 13.9 ± 4.2 | 15.5 % | 34.1 % | 50.4 % |
| Macedonia | | 786 | 25.4 ± 5.0 | 2.3 % | 53.7 % | 28.6 % | 15.4 % |  | 439 | 13.7 ± 5.4 | 14.1 % | 33.5 % | 52.4 % |
| Montenegro | | 330 | 24.4 ± 3.8 | 1.5 % | 63.9 % | 23.9 % | 10.6 % |  | 182 | 15.3 ± 5.4 | 12.6 % | 27.5 % | 59.9 % |
| Poland | | 4 488 | 24.0 ± 4.3 | 3.1 % | 65.1 % | 21.5 % | 10.3 % |  | 2 699 | 14.3 ± 5.4 | 15.7 % | 33.0 % | 51.3 % |
| Romania | | 1 529 | 24.1 ± 4.5 | 4.8 % | 60.9 % | 24.5 % | 9.8 % |  | 888 | 14.6 ± 5.3 | 13.2 % | 33.2 % | 53.6 % |
| Serbia | | 2 082 | 24.7 ± 4.8 | 3.6 % | 56.8 % | 26.7 % | 12.9 % |  | 1 327 | 14.4 ± 5.6 | 14.2 % | 30.1 % | 55.7 % |
| Slovakia | | 160 | 23.9 ± 5.0 | 5.6 % | 61.3 % | 20.6 % | 12.5 % |  | 86 | 13.0 ± 4.7 | 16.3 % | 43.0 % | 40.7 % |
| Slovenia | | 114 | 24.7 ± 5.1 | 2.6 % | 63.2 % | 18.4 % | 15.8 % |  | 65 | 13.6 ± 5.6 | 21.5 % | 24.6 % | 53.8 % |
| **S5 Table, continued.** | | |  |  |  |  |  |  |  |  |  |  |  |
|  |  |  |  |  |  |  |  |  |  |  |  |  |  |
|  | | **Body mass index early in pregnancy** | | | | | |  | **Gestational weight gain**^1^ | | | | |
| **Birth region/county** | | **n** | **BMI (kg/m^2^)** | **Under-weight**  **(%)** | **Normalweight**  **(%)** | **Over-weight**  **(%)** | **Obesity**  **(%)** |  | **n** | **GWG^2^**  **(kg)** | **Inadequate GWG**  **(%)** | **Adequate**  **GWG (%)** | **Excessive GWG**  **(%)** |
| ***Eastern Europe*** | | 4 930 | 23.4 ± 4.1 | 4.8 % | 69.2 % | 19.0 % | 7.0 % |  | 2 963 | 14.3 ± 4.9 | 15.3 % | 36.3 % | 48.4 % |
| Belarus | | 258 | 23.2 ± 4.1 | 5.4 % | 68.6 % | 19.4 % | 6.6 % |  | 158 | 13.7 ± 4.1 | 13.3 % | 44.3 % | 42.4 % |
| Estonia | | 530 | 24.1 ± 4.3 | 3.2 % | 64.7 % | 23.4 % | 8.7 % |  | 307 | 14.9 ± 4.7 | 8.8 % | 37.1 % | 54.1 % |
| Latvia | | 533 | 23.8 ± 4.6 | 2.8 % | 70.2 % | 17.1 % | 9.9 % |  | 329 | 14.5 ± 5.0 | 14.9 % | 33.7 % | 51.4 % |
| Lithuania | | 914 | 23.5 ± 4.0 | 3.5 % | 69.7 % | 20.5 % | 6.3 % |  | 554 | 15.2 ± 5.1 | 11.7 % | 34.5 % | 53.8 % |
| Moldova | | 111 | 23.6 ± 4.5 | 2.7 % | 68.5 % | 22.5 % | 6.3 % |  | 70 | 14.8 ± 3.5 | 8.6 % | 34.3 % | 57.1 % |
| Russia | | 1 814 | 23.2 ± 3.9 | 5.5 % | 70.3 % | 17.7 % | 6.4 % |  | 1 050 | 13.7 ± 4.7 | 19.1 % | 37.7 % | 43.1 % |
| Ukraine | | 770 | 23.1 ± 4.1 | 7.4 % | 68.4 % | 17.8 % | 6.4 % |  | 495 | 14.4 ± 5.2 | 17.0 % | 34.5 % | 48.5 % |
| **High income countries  (Sweden not included)** | | 14 526 | 24.4 ± 4.7 | 2.9 % | 62.9 % | 22.4 % | 11.9 % |  | 7 902 | 13.0 ± 4.8 | 21.1 % | 37.9 % | 41.0 % |
| ***Australasia*** | | 223 | 23.7 ± 4.1 | 0.9 % | 73.5 % | 17.0 % | 8.5 % |  | 128 | 13.0 ± 3.7 | 17.2 % | 43.0 % | 39.8 % |
| Australia | | 181 | 23.7 ± 4.2 | 1.1 % | 72.4 % | 18.2 % | 8.3 % |  | 100 | 13.0 ± 3.7 | 15.0 % | 44.0 % | 41.0 % |
| ***High-income Asia Pacific*** | | 1 037 | 22.7 ± 3.7 | 6.6 % | 72.2 % | 16.1 % | 5.1 % |  | 574 | 11.8 ± 4.3 | 35.5 % | 37.6 % | 26.8 % |
| Japan | | 295 | 21.3 ± 2.8 | 11.5 % | 78.0 % | 9.5 % | 1.0 % |  | 167 | 11.0 ± 3.8 | 47.9 % | 37.7 % | 14.4 % |
| South Korea | | 688 | 23.3 ± 3.8 | 3.8 % | 71.2 % | 18.6 % | 6.4 % |  | 388 | 12.1 ± 4.4 | 30.2 % | 37.6 % | 32.2 % |
| ***High-income North***  ***America*** | | 882 | 24.5 ± 5.1 | 3.2 % | 63.2 % | 22.2 % | 11.5 % |  | 512 | 13.2 ± 4.8 | 20.9 % | 37.7 % | 41.4 % |
| Canada | | 192 | 25.0 ± 5.3 | 2.6 % | 58.9 % | 25.0 % | 13.5 % |  | 101 | 13.1 ± 5.1 | 26.7 % | 30.7 % | 42.6 % |
| USA | | 682 | 24.3 ± 5.1 | 3.4 % | 64.4 % | 21.3 % | 11.0 % |  | 406 | 13.3 ± 4.7 | 19.7 % | 39.2 % | 41.1 % |
| ***Southern Latin***  ***America*** | | 1 608 | 26.9 ± 5.2 | 0.8 % | 40.6 % | 33.0 % | 25.6 % |  | 796 | 13.0 ± 5.7 | 16.3 % | 30.7 % | 53.0 % |
| Argentina | | 168 | 24.4 ± 4.2 | 0.6 % | 63.7 % | 23.8 % | 11.9 % |  | 94 | 12.7 ± 4.6 | 20.2 % | 37.2 % | 42.6 % |
| Chile | | 1 373 | 27.4 ± 5.3 | 0.7 % | 36.9 % | 34.5 % | 28.0 % |  | 672 | 13.0 ± 5.8 | 15.8 % | 29.6 % | 54.6 % |
|  | |  |  |  |  |  |  |  |  |  |  |  |  |
| **S5 Table, continued.** | | |  |  |  |  |  |  |  |  |  |  |  |
|  |  |  |  |  |  |  |  |  |  |  |  |  |  |
|  | | **Body mass index early in pregnancy** | | | | | |  | **Gestational weight gain**^1^ | | | | |
| **Birth region/county** | | **n** | **BMI (kg/m^2^)** | **Under-weight**  **(%)** | **Normalweight**  **(%)** | **Over-weight**  **(%)** | **Obesity**  **(%)** |  | **n** | **GWG^2^**  **(kg)** | **Inadequate GWG**  **(%)** | **Adequate**  **GWG (%)** | **Excessive GWG**  **(%)** |
| ***Western Europe*** | | 10 776 | 24.2 ± 4.5 | 2.8 % | 65.1 % | 21.5 % | 10.6 % |  | 5 892 | 13.1 ± 4.8 | 20.4 % | 38.8 % | 40.8 % |
| Austria | | 172 | 23.5 ± 4.1 | 1.7 % | 72.1 % | 18.0 % | 8.1 % |  | 87 | 12.8 ± 4.3 | 23.0 % | 37.9 % | 39.1 % |
| Belgium | | 128 | 22.9 ± 3.8 | 7.8 % | 65.6 % | 21.1 % | 5.5 % |  | 68 | 13.0 ± 5.7 | 26.5 % | 36.8 % | 36.8 % |
| Denmark | | 1 161 | 25.1 ± 5.3 | 3.4 % | 57.0 % | 23.9 % | 15.7 % |  | 688 | 13.0 ± 5.4 | 20.8 % | 33.4 % | 45.8 % |
| Finland | | 1 962 | 24.4 ± 4.7 | 1.8 % | 64.8 % | 21.8 % | 11.7 % |  | 984 | 12.9 ± 4.6 | 20.7 % | 40.8 % | 38.5 % |
| France | | 501 | 23.2 ± 4.2 | 3.4 % | 73.9 % | 14.6 % | 8.2 % |  | 282 | 12.6 ± 4.2 | 23.0 % | 43.3 % | 33.7 % |
| Germany | | 2 051 | 23.9 ± 4.2 | 3.2 % | 66.7 % | 21.5 % | 8.5 % |  | 1 072 | 13.2 ± 4.8 | 19.0 % | 40.2 % | 40.8 % |
| Greece | | 611 | 24.6 ± 4.7 | 1.8 % | 60.6 % | 24.7 % | 12.9 % |  | 369 | 14.1 ± 5.2 | 17.3 % | 32.0 % | 50.7 % |
| Iceland | | 421 | 24.4 ± 4.1 | 1.4 % | 62.7 % | 24.9 % | 10.9 % |  | 241 | 12.7 ± 4.6 | 21.6 % | 41.9 % | 36.5 % |
| Ireland | | 94 | 24.2 ± 5.1 | 1.1 % | 73.4 % | 17.0 % | 8.5 % |  | 59 | 12.9 ± 4.9 | 15.3 % | 62.7 % | 22.0 % |
| Israel | | 150 | 25.1 ± 4.9 | 4.0 % | 54.0 % | 27.3 % | 14.7 % |  | 89 | 13.3 ± 4.5 | 14.6 % | 33.7 % | 51.7 % |
| Italy | | 368 | 23.3 ± 4.0 | 4.6 % | 71.7 % | 16.8 % | 6.8 % |  | 226 | 12.9 ± 3.7 | 22.6 % | 42.9 % | 34.5 % |
| Netherlands | | 346 | 24.0 ± 4.5 | 2.6 % | 66.5 % | 22.0 % | 9.0 % |  | 192 | 12.5 ± 4.5 | 22.9 % | 41.7 % | 35.4 % |
| Norway | | 1 126 | 24.3 ± 4.5 | 1.9 % | 65.5 % | 21.5 % | 11.2 % |  | 559 | 13.4 ± 4.8 | 19.3 % | 37.4 % | 43.3 % |
| Portugal | | 135 | 23.4 ± 3.9 | 5.2 % | 68.1 % | 17.0 % | 9.6 % |  | 82 | 13.5 ± 5.1 | 18.3 % | 41.5 % | 40.2 % |
| Spain | | 579 | 23.5 ± 4.0 | 4.3 % | 67.2 % | 20.9 % | 7.6 % |  | 322 | 12.9 ± 4.9 | 24.5 % | 40.1 % | 35.4 % |
| Switzerland | | 140 | 23.1 ± 3.6 | 4.3 % | 75.0 % | 15.0 % | 5.7 % |  | 77 | 13.1 ± 4.7 | 31.2 % | 35.1 % | 33.8 % |
| UK | | 646 | 24.2 ± 4.5 | 3.3 % | 65.2 % | 20.9 % | 10.7 % |  | 387 | 13.3 ± 4.8 | 18.6 % | 35.1 % | 46.3 % |
| **Latin America and**  **Caribbean** | | 3 274 | 25.1 ± 4.5 | 2.2 % | 55.1 % | 29.2 % | 13.5 % |  | 1 843 | 12.5 ± 4.8 | 21.9 % | 35.6 % | 42.5 % |
| ***Andean Latin***  ***America*** | | 873 | 25.1 ± 4.1 | 1.5 % | 54.9 % | 30.8 % | 12.8 % |  | 508 | 11.8 ± 4.5 | 25.2 % | 40.0 % | 34.8 % |
| Bolivia | | 243 | 25.4 ± 4.1 | 0.8 % | 51.4 % | 33.3 % | 14.4 % |  | 140 | 12.8 ± 5.2 | 20.0 % | 39.3 % | 40.7 % |
| Ecuador | | 138 | 25.4 ± 4.5 | 1.4 % | 54.3 % | 29.7 % | 14.5 % |  | 85 | 11.3 ± 4.5 | 25.9 % | 37.6 % | 36.5 % |
| Peru | | 492 | 25.0 ± 4.1 | 1.8 % | 56.7 % | 29.9 % | 11.6 % |  | 283 | 11.5 ± 4.1 | 27.6 % | 41.0 % | 31.4 % |
| ***Caribbean*** | | 251 | 24.6 ± 4.2 | 3.2 % | 57.0 % | 29.9 % | 10.0 % |  | 143 | 13.6 ± 5.5 | 19.6 % | 24.5 % | 55.9 % |
| Cuba | | 139 | 24.4 ± 4.1 | 2.9 % | 61.2 % | 28.1 % | 7.9 % |  | 84 | 13.4 ± 5.8 | 20.2 % | 26.2 % | 53.6 % |
| **S5 Table, continued.** | | |  |  |  |  |  |  |  |  |  |  |  |
|  |  |  |  |  |  |  |  |  |  |  |  |  |  |
|  | | **Body mass index early in pregnancy** | | | | | |  | **Gestational weight gain**^1^ | | | | |
| **Birth region/county** | | **n** | **BMI (kg/m^2^)** | **Under-weight**  **(%)** | **Normalweight**  **(%)** | **Over-weight**  **(%)** | **Obesity**  **(%)** |  | **n** | **GWG^2^**  **(kg)** | **Inadequate GWG**  **(%)** | **Adequate**  **GWG**  **(%)** | **Excessive GWG**  **(%)** |
| ***Central Latin America*** | | 1 506 | 25.4 ± 4.8 | 1.4 % | 54.9 % | 28.1 % | 15.6 % |  | 852 | 12.4 ± 4.9 | 21.1 % | 35.0 % | 43.9 % |
| Colombia | | 796 | 25.4 ± 4.8 | 1.5 % | 56.4 % | 27.1 % | 14.9 % |  | 458 | 12.8 ± 4.9 | 18.3 % | 34.1 % | 47.6 % |
| El Salvador | | 207 | 26.6 ± 4.4 | 0 % | 41.1 % | 39.1 % | 19.8 % |  | 103 | 11.8 ± 5.3 | 20.4 % | 35.9 % | 43.7 % |
| Mexico | | 196 | 24.3 ± 4.7 | 1.5 % | 67.9 % | 20.4 % | 10.2 % |  | 113 | 11.7 ± 3.8 | 25.7 % | 42.5 % | 31.9 % |
| Venezuela | | 104 | 25.4 ± 4.8 | 3.8 % | 51.0 % | 26.9 % | 18.3 % |  | 69 | 13.5 ± 4.6 | 13.0 % | 29.0 % | 58.0 % |
| ***Tropical Latin***  ***America*** | | 644 | 24.5 ± 4.2 | 4.5 % | 55.3 % | 29.2 % | 11.0 % |  | 340 | 13.1 ± 4.6 | 19.7 % | 35.3 % | 45.0 % |
| Brazil | | 623 | 24.4 ± 4.2 | 4.7 % | 55.7 % | 29.1 % | 10.6 % |  | 330 | 13.1 ± 4.5 | 19.4 % | 35.8 % | 44.8 % |
| **North Africa and**  **Middle East** | | 35 089 | 25.5 ± 4.6 | 2.2 % | 49.8 % | 32.6 % | 15.3 % |  | 20 757 | 13.2 ± 5.4 | 17.4 % | 32.5 % | 50.1 % |
| Afghanistan | | 2 311 | 25.0 ± 4.4 | 3.8 % | 51.8 % | 31.8 % | 12.6 % |  | 1 508 | 12.7 ± 5.0 | 20.1 % | 35.7 % | 44.2 % |
| Algeria | | 268 | 26.2 ± 4.5 | 2.2 % | 39.6 % | 39.9 % | 18.3 % |  | 157 | 12.1 ± 5.6 | 25.5 % | 26.1 % | 48.4 % |
| Egypt | | 484 | 27.1 ± 5.0 | 1.7 % | 36.2 % | 36.6 % | 25.6 % |  | 287 | 11.3 ± 5.3 | 24.0 % | 32.1 % | 43.9 % |
| Iran | | 3 754 | 24.5 ± 4.1 | 1.9 % | 60.7 % | 27.3 % | 10.1 % |  | 2 166 | 14.4 ± 5.2 | 13.1 % | 32.5 % | 54.4 % |
| Iraq | | 11 748 | 25.8 ± 4.5 | 1.9 % | 46.5 % | 35.2 % | 16.4 % |  | 6 328 | 13.2 ± 5.3 | 16.2 % | 32.8 % | 51.0 % |
| Jordan | | 479 | 25.8 ± 4.7 | 1.9 % | 50.1 % | 30.5 % | 17.5 % |  | 266 | 12.3 ± 5.4 | 22.9 % | 35.7 % | 41.4 % |
| Kuwait | | 130 | 26.2 ± 5.6 | 2.3 % | 50.0 % | 26.9 % | 20.8 % |  | 78 | 12.4 ± 5.4 | 24.4 % | 28.2 % | 47.4 % |
| Lebanon | | 2 209 | 25.6 ± 4.9 | 2.3 % | 50.6 % | 30.6 % | 16.6 % |  | 1 143 | 13.4 ± 5.3 | 16.7 % | 32.1 % | 51.2 % |
| Libya | | 184 | 25.8 ± 4.3 | 1.6 % | 47.3 % | 36.4 % | 14.7 % |  | 110 | 12.6 ± 5.6 | 25.5 % | 26.4 % | 48.2 % |
| Morocco | | 1 171 | 25.5 ± 4.1 | 2.7 % | 45.2 % | 37.5 % | 14.6 % |  | 629 | 11.7 ± 5.5 | 28.0 % | 32.8 % | 39.3 % |
| Palestine | | 731 | 26.0 ± 5.7 | 4.7 % | 46.5 % | 28.9 % | 20.0 % |  | 375 | 12.3 ± 5.5 | 21.1 % | 35.5 % | 43.5 % |
| Saudi Arabia | | 244 | 25.2 ± 4.5 | 3.7 % | 49.2 % | 32.4 % | 14.8 % |  | 150 | 12.2 ± 5.7 | 20.0 % | 32.7 % | 47.3 % |
| Sudan | | 337 | 25.3 ± 4.7 | 7.7 % | 41.8 % | 35.6 % | 14.8 % |  | 215 | 10.9 ± 5.4 | 29.8 % | 34.0 % | 36.3 % |
| Syria | | 6 582 | 25.5 ± 4.8 | 2.0 % | 51.3 % | 30.6 % | 16.1 % |  | 4 812 | 13.0 ± 5.4 | 18.6 % | 32.5 % | 48.9 % |
| Tunisia | | 454 | 25.8 ± 4.5 | 3.1 % | 43.8 % | 36.8 % | 16.3 % |  | 275 | 12.7 ± 4.9 | 18.5 % | 32.7 % | 48.7 % |
| Turkey | | 3 733 | 25.4 ± 4.4 | 1.8 % | 51.1 % | 33.2 % | 13.9 % |  | 2 103 | 14.3 ± 5.5 | 11.5 % | 29.7 % | 58.9 % |
| Yemen | | 167 | 25.2 ± 4.7 | 5.4 % | 49.7 % | 30.5 % | 14.4 % |  | 88 | 10.7 ± 5.1 | 34.1 % | 31.8 % | 34.1 % |
| **S5 Table, continued.** | | |  |  |  |  |  |  |  |  |  |  |  |
|  |  |  |  |  |  |  |  |  |  |  |  |  |  |
|  | | **Body mass index early in pregnancy** | | | | | |  | **Gestational weight gain**^1^ | | | | |
| **Birth region/county** | | **n** | **BMI (kg/m^2^)** | **Under-weight**  **(%)** | **Normalweight**  **(%)** | **Over-weight**  **(%)** | **Obesity**  **(%)** |  | **n** | **GWG^2^**  **(kg)** | **Inadequate GWG**  **(%)** | **Adequate**  **GWG (%)** | **Excessive GWG**  **(%)** |
| **South Asia** | | 4 337 | 24.7 ± 4.5 | 4.1 % | 54.4 % | 29.1 % | 12.3 % |  | 2 510 | 12.1 ± 5.0 | 24.9 % | 34.9 % | 40.2 % |
| Bangladesh | | 721 | 24.9 ± 4.2 | 2.5 % | 54.6 % | 31.1 % | 11.8 % |  | 418 | 12.6 ± 5.1 | 22.2 % | 33.3 % | 44.5 % |
| India | | 2 050 | 24.5 ± 4.4 | 4.6 % | 56.2 % | 26.9 % | 12.2 % |  | 1 091 | 11.8 ± 4.9 | 26.4 % | 37.1 % | 36.5 % |
| Pakistan | | 1 494 | 24.9 ± 4.6 | 4.1 % | 51.8 % | 31.5 % | 12.6 % |  | 946 | 12.3 ± 5.1 | 23.5 % | 33.6 % | 42.9 % |
| **Southeast Asia and**  **East Asia** | | 8 547 | 22.7 ± 3.7 | 8.3 % | 69.9 % | 17.1 % | 4.6 % |  | 4 621 | 13.1 ± 4.4 | 20.8 % | 43.1 % | 36.2 % |
| ***East Asia*** | | 2 317 | 21.7 ± 3.0 | 10.5 % | 77.2 % | 10.9 % | 1.4 % |  | 1 422 | 13.3 ± 4.0 | 21.0 % | 45.8 % | 33.2 % |
| China | | 2 233 | 21.7 ± 2.9 | 10.6 % | 77.1 % | 11.0 % | 1.3 % |  | 1 374 | 13.3 ± 3.9 | 21.0 % | 45.8 % | 33.3 % |
| ***Southeast Asia*** | | 6 230 | 23.1 ± 3.9 | 7.5 % | 67.2 % | 19.5 % | 5.8 % |  | 3 199 | 13.0 ± 4.6 | 20.6 % | 41.9 % | 37.5 % |
| Indonesia | | 247 | 24.0 ± 3.7 | 4.5 % | 58.7 % | 31.6 % | 5.3 % |  | 127 | 11.9 ± 4.9 | 24.4 % | 44.9 % | 30.7 % |
| Philippines | | 1 199 | 23.4 ± 3.6 | 6.2 % | 64.7 % | 23.6 % | 5.5 % |  | 610 | 12.6 ± 4.6 | 23.9 % | 39.8 % | 36.2 % |
| Sri Lanka | | 818 | 24.4 ± 4.8 | 6.4 % | 54.9 % | 27.9 % | 10.9 % |  | 386 | 12.1 ± 4.8 | 20.5 % | 41.2 % | 38.3 % |
| Thailand | | 2 661 | 23.1 ± 4.0 | 7.7 % | 67.6 % | 18.5 % | 6.2 % |  | 1 347 | 13.7 ± 4.7 | 16.6 % | 40.7 % | 42.7 % |
| Vietnam | | 1 076 | 21.5 ± 2.6 | 9.7 % | 81.4 % | 7.7 % | 1.2 % |  | 584 | 12.9 ± 3.9 | 25.5 % | 44.5 % | 30.0 % |
| **Sub-Saharan Africa** | | 13 813 | 25.9 ± 5.2 | 5.0 % | 43.3 % | 31.7 % | 20.0 % |  | 8 680 | 10.3 ± 5.5 | 34.7 % | 34.9 % | 30.4 % |
| ***Central  sub-Saharan***  ***Africa*** | | 441 | 27.2 ± 5.1 | 0.9 % | 34.2 % | 37.2 % | 27.7 % |  | 246 | 11.4 ± 6.3 | 26.4 % | 29.3 % | 44.3 % |
| Democratic  Republic of the  Congo | | 382 | 27.4 ± 5.0 | 0.8 % | 32.2 % | 38.2 % | 28.8 % |  | 219 | 11.1 ± 6.3 | 28.3 % | 28.8 % | 42.9 % |
| **S5 Table, continued.** | | |  |  |  |  |  |  |  |  |  |  |  |
|  |  |  |  |  |  |  |  |  |  |  |  |  |  |
|  | | **Body mass index early in pregnancy** | | | | | |  | **Gestational weight gain**^1^ | | | | |
| **Birth region/county** | | **n** | **BMI (kg/m^2^)** | **Under-weight**  **(%)** | **Normalweight**  **(%)** | **Over-weight**  **(%)** | **Obesity**  **(%)** |  | **n** | **GWG^2^**  **(kg)** | **Inadequate GWG**  **(%)** | **Adequate**  **GWG (%)** | **Excessive GWG**  **(%)** |
| ***Eastern***  ***Sub-Saharan Africa*** | | 11 513 | 25.7 ± 5.3 | 5.5 % | 44.3 % | 30.8 % | 19.4 % |  | 7 328 | 10.1 ± 5.5 | 36.5 % | 35.5 % | 28.0 % |
| Burundi | | 355 | 26.2 ± 5.2 | 3.7 % | 40.3 % | 37.2 % | 18.9 % |  | 187 | 10.5 ± 4.9 | 31.0 % | 33.7 % | 35.3 % |
| Eritrea | | 2 456 | 23.4 ± 4.2 | 10.1 % | 60.0 % | 22.6 % | 7.3 % |  | 1 686 | 12.2 ± 4.7 | 29.2 % | 38.8 % | 32.0 % |
| Ethiopia | | 1 127 | 24.1 ± 4.1 | 5.7 % | 57.4 % | 28.5 % | 8.4 % |  | 677 | 12.6 ± 5.0 | 23.8 % | 37.8 % | 38.4 % |
| Kenya | | 297 | 24.9 ± 4.4 | 3.4 % | 54.2 % | 29.3 % | 13.1 % |  | 181 | 11.3 ± 5.8 | 32.0 % | 28.2 % | 39.8 % |
| Somalia | | 6 666 | 26.9 ± 5.5 | 4.3 % | 35.7 % | 33.7 % | 26.3 % |  | 4 256 | 8.8 ± 5.4 | 42.6 % | 34.2 % | 23.2 % |
| Tanzania | | 136 | 25.3 ± 4.5 | 2.2 % | 48.5 % | 33.8 % | 15.4 % |  | 80 | 10.7 ± 5.0 | 31.3 % | 30.0 % | 38.8 % |
| Uganda | | 291 | 26.0 ± 5.2 | 2.1 % | 48.8 % | 28.9 % | 20.3 % |  | 161 | 12.4 ± 5.4 | 22.4 % | 34.2 % | 43.5 % |
| ***Southern***  ***Sub-Saharan Africa*** | | 121 | 24.7 ± 4.4 | 3.3 % | 58.7 % | 28.1 % | 9.9 % |  | 64 | 13.2 ± 4.9 | 17.2 % | 37.5 % | 45.3 % |
| ***Western***  ***Sub-Saharan Africa*** | | 1 738 | 26.7 ± 5.0 | 2.5 % | 37.6 % | 37.2 % | 22.8 % |  | 1 042 | 11.1 ± 5.6 | 25.1 % | 31.9 % | 43.0 % |
| Cameroon | | 163 | 27.5 ± 4.7 | 1.2 % | 31.3 % | 37.4 % | 30.1 % |  | 107 | 12.7 ± 6.0 | 12.1 % | 33.6 % | 54.2 % |
| The Gambia | | 385 | 26.3 ± 5.3 | 3.6 % | 41.8 % | 33.2 % | 21.3 % |  | 216 | 10.0 ± 5.9 | 36.1 % | 29.6 % | 34.3 % |
| Ghana | | 253 | 26.9 ± 4.9 | 1.6 % | 34.8 % | 42.3 % | 21.3 % |  | 160 | 11.7 ± 5.2 | 20.6 % | 30.0 % | 49.4 % |
| Nigeria | | 456 | 27.2 ± 4.5 | 1.1 % | 33.6 % | 40.8 % | 24.6 % |  | 274 | 11.2 ± 5.1 | 20.8 % | 33.9 % | 45.3 % |
| Sierra Leone | | 96 | 25.9 ± 5.1 | 2.1 % | 49.0 % | 29.2 % | 19.8 % |  | 48 | 12.2 ± 5.5 | 18.8 % | 31.3 % | 50.0 % |

Data are presented as mean ± standard deviations or as %.

BMI, body mass index; GWG, gestational weight gain.

^1^ GWG as below (inadequate), within (adequate) or above (excessive) the National Academy of Medicine (formerly Institute of Medicine) recommendations.

^2^ Calculated as the last recorded weight in pregnancy minus weight at first visit in antenatal care.
