# Supplementary material for: Body mass index and gestational weight gain in migrant women by birth regions compared with Swedish-born women: A registry linkage study of 0.5 million pregnancies
Source: PLoS One. 2020 Oct 29;15(10):e0241319. doi: 10.1371/journal.pone.0241319 (PMC7595374; doi:10.1371/journal.pone.0241319)
Supplement: S6 Table — (DOCX) [file pone.0241319.s009.docx]

**S6 Table.** Availability of data in the database retrieved from the Swedish Pregnancy Register (2010-2018) stratified by birth region.

|  | **n** | **Available**  **BMI data** | **Missing data on gestational age at first visit in maternity care** | **Weight not recorded in first trimester^1^** | **Missing data on education** | **Missing data on parity** | **Women included in the  main analysis** |
| --- | --- | --- | --- | --- | --- | --- | --- |
| All women | 841 503 | 94.4 % | 11.5 % | 23.2 % | 18.6 % | 2.3 % | 63.6 % |
| Sweden | 577 919 | 95.1 % | 5.3 % | 15.5 % | 10.1 % | 1.9 % | 74.7 % |
| Central Europe, Eastern Europe and Central Asia | 38 450 | 95.7 % | 6.0 % | 23.8 % | 18.4 % | 1.0 % | 62.5 % |
| High income countries | 20 952 | 95.0 % | 6.4 % | 20.0 % | 11.8 % | 1.3 % | 69.3 % |
| Latin America and Caribbean | 5 150 | 95.6 % | 6.5 % | 22.6 % | 18.0 % | 1.5 % | 63.6 % |
| North Africa and  Middle East | 59 818 | 95.5 % | 6.2 % | 23.3 % | 23.5 % | 0.8 % | 58.7 % |
| South Asia | 6 898 | 95.3 % | 6.5 % | 23.2 % | 17.9 % | 0.9 % | 62.9 % |
| Southeast Asia and  East Asia | 14 503 | 96.0 % | 5.6 % | 23.6 % | 23.4 % | 1.1 % | 58.9 % |
| Sub-Saharan Africa | 31 223 | 95.2 % | 6.0 % | 37.2 % | 31.1 % | 0.7 % | 44.2 % |
| No reported birth country | 86 590 | 87.7 % | 63.5 % | 69.5 % | 68.7 % | 7.6 % |  |

^1^ this percentage also include women with missing data on gestational age at first visit in antenatal care.
