# Supplementary material for: Body mass index and gestational weight gain in migrant women by birth regions compared with Swedish-born women: A registry linkage study of 0.5 million pregnancies
Source: PLoS One. 2020 Oct 29;15(10):e0241319. doi: 10.1371/journal.pone.0241319 (PMC7595374; doi:10.1371/journal.pone.0241319)
Supplement: S7 Table — (DOCX) [file pone.0241319.s010.docx]

**S7 Table.** Comparing the prevalence and unadjusted odds ratios of obesity and underweight by birth regions with all available data as compared to the analytic sample.

|  | **Obesity** | | | |  | **Underweight** | | | |
| --- | --- | --- | --- | --- | --- | --- | --- | --- | --- |
|  | ***Analytic sample***  ***(n = 535 609)*** | | ***All available data***  ***(n =718 677)*** | |  | ***Analytic sample***  ***(n = 535 609)*** | | ***All available data***  ***(n =718 677)*** | |
| **Birth region** | **Prevalence** | **OR**  **(95 % CI)** | **Prevalence** | **OR**  **(95 % CI)** |  | **Prevalence** | **OR**  **(95 % CI)** | **Prevalence** | **OR**  **(95 % CI)** |
| Sweden | 12.7 % | Reference | 13.0 % | Reference |  | 2.3 % | Reference | 2.2 % | Reference |
| Central Europe, Eastern Europe and Central Asia | 10.1 % | 0.76  (0.73-0.80) | 10.8 % | 0.81  (0.78-0.84) |  | 3.6 % | 1.55  (1.44-1.66) | 3.6 % | 1.55  (1.47-1.65) |
| High income countries | 11.9 % | 0.90  (0.86-0.95) | 12.3 % | 0.92  (0.88-0.96) |  | 2.9 % | 1.23  (1.11-1.35) | 2.7 % | 1.18  (1.09-1.29) |
| Latin America and Caribbean | 13.5 % | 1.17  (1.05-1.30) | 13.5 % | 1.14  (1.05-1.25) |  | 2.2 % | 1.06  (0.83-1.34) | 2.0 % | 0.97  (0.79-1.19) |
| North Africa and Middle East | 15.3 % | 1.47  (1.42-1.51) | 17.1 % | 1.67  (1.62-1.71) |  | 2.2 % | 1.19  (1.11-1.29) | 2.1 % | 1.17  (1.10-1.25) |
| South Asia | 12.3 % | 1.08  (0.98-1.19) | 12.8 % | 1.12  (1.04-1.21) |  | 4.1 % | 2.04  (1.75-2.37) | 3.8 % | 1.95  (1.71-2.22) |
| Southeast Asia and East Asia | 4.7 % | 0.32  (0.29-0.35) | 4.7 % | 0.31  (0.29-0.34) |  | 8.3 % | 3.20  (2.95-3.47) | 7.9 % | 3.04  (2.85-3.25) |
| Sub-Saharan Africa | 20.0 % | 2.21  (2.11-2.31) | 22.7 % | 2.59  (2.51-2.67) |  | 5.0 % | 3.07  (2.83-3.33) | 4.4 % | 2.88  (2.71-3.06) |
