# Supplementary material for: Body mass index and gestational weight gain in migrant women by birth regions compared with Swedish-born women: A registry linkage study of 0.5 million pregnancies
Source: PLoS One. 2020 Oct 29;15(10):e0241319. doi: 10.1371/journal.pone.0241319 (PMC7595374; doi:10.1371/journal.pone.0241319)
Supplement: S8 Table — (DOCX) [file pone.0241319.s011.docx]

**S8 Table.** Comparing the prevalence and unadjusted odds ratios of excessive and inadequate gestational weight gain (GWG) by birth regions with all available data as compared to the analytic sample.

|  | **Excessive gestational weight gain** | | | |  | **Inadequate gestational weight gain** | | | |
| --- | --- | --- | --- | --- | --- | --- | --- | --- | --- |
|  | ***Analytic sample***  ***(n =270 044)*** | | ***All available data***  ***(n = 294 437)*** | |  | ***Analytic sample***  ***(n = 270 044)*** | | ***All available data***  ***(n = 294 437)*** | |
| **Birth region** | **Prevalence** | **OR**  **(95 % CI)** | **Prevalence** | **OR**  **(95 % CI)** |  | **Prevalence** | **OR**  **(95 % CI)** | **Prevalence** | **OR**  **(95 % CI)** |
| Sweden | 47.4 % | Reference | 47.5 % | Reference |  | 17.5 % | Reference | 17.5 % | Reference |
| Central Europe, Eastern Europe and Central Asia | 52.4 % | 1.17  (1.13-1.22) | 52.2 % | 1.16  (1.12-1.20) |  | 14.5 % | 0.88  (0.83-0.93) | 14.5 % | 0.88  (0.84-0.93) |
| High income countries | 41.0 % | 0.80  (0.76-0.84) | 41.2 % | 0.80  (0.77-0.84) |  | 21.1 % | 1.12  (1.05-1.19) | 21.0 % | 1.11  (1.05-1.18) |
| Latin America and Caribbean | 42.5 % | 0.89  (0.80-0.98) | 42.6 % | 0.88  (0.80-0.97) |  | 21.9 % | 1.24  (1.09-1.40) | 21.7 % | 1.22  (1.08-1.37) |
| North Africa and Middle East | 50.1 % | 1.14  (1.11-1.18) | 49.7 % | 1.13  (1.09-1.16) |  | 17.4 % | 1.07  (1.03-1.12) | 17.7 % | 1.09  (1.05-1.13) |
| South Asia | 40.2 % | 0.85  (0.78-0.94) | 40.1 % | 0.85  (0.78-0.92) |  | 24.9 % | 1.44  (1.30-1.59) | 24.8 % | 1.42  (1.29-1.56) |
| Southeast Asia and East Asia | 36.2 % | 0.62  (0.58-0.67) | 36.3 % | 0.62  (0.59-0.66) |  | 20.8 % | 0.97  (0.90-1.05) | 20.8 % | 0.97  (0.90-1.04) |
| Sub-Saharan Africa | 30.4 % | 0.65  (0.61-0.68) | 29.5 % | 0.63  (0.60-0.66) |  | 34.7 % | 2.00  (1.90-2.11) | 35.9 % | 2.08  (1.99-2.18) |
