# Supplementary material for: Body mass index and gestational weight gain in migrant women by birth regions compared with Swedish-born women: A registry linkage study of 0.5 million pregnancies
Source: PLoS One. 2020 Oct 29;15(10):e0241319. doi: 10.1371/journal.pone.0241319 (PMC7595374; doi:10.1371/journal.pone.0241319)
Supplement: S9 Table — (DOCX) [file pone.0241319.s012.docx]

**S9 Table.** Comparing the odds ratios of excessive and inadequate gestational weight gain (GWG) by birth regions, using two GWG calculations.

|  | **Excessive GWG calculated using original calculation^1^**  **(main results)** | | |  | **Excessive GWG calculated using the recommended gains for the second and third trimester^2^** | | |
| --- | --- | --- | --- | --- | --- | --- | --- |
|  | **Prevalence** | **OR (95 % CI)** | **OR (95 % CI)** |  | **Prevalence** | **OR (95 % CI)** | **OR (95 % CI)** |
| **Birth region** |  | ***Basic adjustment*** ^3^ | ***Basic adjustment*** ^3^ ***+ education*** |  |  | ***Basic adjustment*** ^3^ | ***Basic adjustment*** ^3^ ***+ education*** |
| Sweden | 47.4 % | Reference | Reference |  | 67.3 % | Reference | Reference |
| Central Europe, Eastern Europe and Central Asia | 52.4 % | 1.18 (1.14-1.23) | 1.16 (1.12-1.21) |  | 72.1 % | 1.27 (1.21-1.32) | 1.25 (1.19-1.30) |
| High income countries | 41.0 % | 0.84 (0.79-0.88) | 0.86 (0.82-0.91) |  | 61.9 % | 0.82 (0.78-0.87) | 0.85 (0.80-0.89) |
| Latin America and Caribbean | 42.5 % | 0.92 (0.83-1.02) | 0.89 (0.80-0.99) |  | 63.0 % | 0.94 (0.84-1.05) | 0.92 (0.82-1.02) |
| North Africa and Middle East | 50.1 % | 1.15 (1.11-1.18) | 1.11 (1.08-1.15) |  | 68.9 % | 1.18 (1.14-1.22) | 1.15 (1.11-1.20) |
| South Asia | 40.2 % | 0.84 (0.77-0.92) | 0.87 (0.79-0.95) |  | 58.7 % | 0.81 (0.74-0.89) | 0.84 (0.76-0.92) |
| Southeast Asia and East Asia | 36.2 % | 0.64 (0.60-0.68) | 0.62 (0.58-0.66) |  | 60.1 % | 0.75 (0.70-0.80) | 0.73 (0.68-0.78) |
| Sub-Saharan Africa | 30.4 % | 0.67 (0.64-0.71) | 0.62 (0.59-0.66) |  | 48.2 % | 0.68 (0.65-0.72) | 0.64 (0.61-0.68) |

|  | **Inadequate GWG using original calculation^1^**  **(main results)** | | |  | **Inadequate GWG calculated using the recommended gains for the second and third trimester^2^** | | |
| --- | --- | --- | --- | --- | --- | --- | --- |
|  | **Prevalence** | **OR (95 % CI)** | **OR (95 % CI)** |  | **Prevalence** | **OR (95 % CI)** | **OR (95 % CI)** |
| **Birth region** |  | ***Basic adjustment*** ^3^ | ***Basic adjustment*** ^3^ ***+ education*** |  |  | ***Basic adjustment*** ^3^ | ***Basic adjustment*** ^3^ ***+ education*** |
| Sweden | 17.5 % | Reference | Reference |  | 9.7 % | Reference | Reference |
| Central Europe, Eastern Europe and Central Asia | 14.5 % | 0.87 (0.83-0.92) | 0.85 (0.81-0.90) |  | 8.3 % | 1.00 (0.93-1.07) | 0.95 (0.88-1.02) |
| High income countries | 21.1 % | 1.13 (1.06-1.20) | 1.12 (1.06-1.20) |  | 11.5 % | 1.05 (0.97-1.14) | 1.07 (0.99-1.16) |
| Latin America and Caribbean | 21.9 % | 1.24 (1.09-1.40) | 1.21 (1.07-1.37) |  | 13.5 % | 1.38 (1.18-1.61) | 1.30 (1.11-1.52) |
| North Africa and Middle East | 17.4 % | 1.06 (1.02-1.11) | 0.99 (0.95-1.04) |  | 11.1 % | 1.28 (1.21-1.35) | 1.12 (1.06-1.18) |
| South Asia | 24.9 % | 1.44 (1.30-1.60) | 1.42 (1.28-1.58) |  | 16.9 % | 1.65 (1.45-1.86) | 1.64 (1.44-1.86) |
| Southeast Asia and East Asia | 20.8 % | 0.97 (0.90-1.05) | 0.92 (0.85-1.00) |  | 11.9 % | 1.02 (0.92-1.13) | 0.90 (0.82-1.00) |
| Sub-Saharan Africa | 34.7 % | 1.97 (1.87-2.07) | 1.73 (1.63-1.83) |  | 26.7 % | 2.48 (2.33-2.63) | 1.92 (1.80-2.05) |

^1^ GWG recommendations for the entire pregnancy was tailored to classify GWG as excessive, adequate and inadequate [3] (see S1 Supplementary Methods).

^2^ GWG was divided by number of weeks in second and third trimester which was utilized to classify GWG as excessive, adequate and inadequate [3].

^3^ Basic adjustments in the analyses were age, parity and gestational age at first antenatal care visit.
