# Supplementary material for: Body mass index and gestational weight gain in migrant women by birth regions compared with Swedish-born women: A registry linkage study of 0.5 million pregnancies
Source: PLoS One. 2020 Oct 29;15(10):e0241319. doi: 10.1371/journal.pone.0241319 (PMC7595374; doi:10.1371/journal.pone.0241319)
Supplement: S10 Table — (DOCX) [file pone.0241319.s013.docx]

**S10 Table.** Comparing the odds ratios of excessive and inadequate gestational weight gain (GWG) in normal weight women (n = 160 658) using the original calculation as compared to the cut-offs by INTERGROWTH-21.

|  | **Excessive GWG in normal weight women using original calculation (main results)** | | |  | **Excessive GWG in normal weight women using  INTERGROWTH-21 cut-offs^1^** | | |
| --- | --- | --- | --- | --- | --- | --- | --- |
|  | **Prevalence** | **OR (95 % CI)** | **OR (95 % CI)** |  | **Prevalence** | **OR (95 % CI)** | **OR (95 % CI)** |
| **Birth region** |  | ***Basic adjustment***^2^ | ***Basic adjustmen***^2^ ***+ education*** |  |  | ***Basic adjustment***^2^ | ***Basic adjustment***^2^ ***+ education*** |
| Sweden | 35.1 % | Reference | Reference |  | 22.8 % | Reference | Reference |
| Central Europe, Eastern Europe and Central Asia | 43.7 % | 1.38 (1.32-1.45) | 1.34 (1.28-1.41) |  | 30.1 % | 1.45 (1.38-1.52) | 1.41 (1.34-1.48) |
| High income countries | 29.4 % | 0.85 (0.80-0.91) | 0.88 (0.82-0.94) |  | 17.7 % | 0.79 (0.73-0.85) | 0.82 (0.76-0.88) |
| Latin America and Caribbean | 28.3 % | 0.82 (0.71-0.96) | 0.80 (0.69-0.93) |  | 17.2 % | 0.77 (0.65-0.91) | 0.74 (0.63-0.87) |
| North Africa and Middle East | 39.2 % | 1.22 (1.16-1.28) | 1.17 (1.12-1.23) |  | 26.9 % | 1.23 (1.18-1.29) | 1.18 (1.12-1.24) |
| South Asia | 30.4 % | 0.95 (0.83-1.08) | 0.97 (0.85-1.10) |  | 19.2 % | 0.87 (0.75-1.00) | 0.90 (0.78-1.03) |
| Southeast Asia and East Asia | 32.2 % | 0.91 (0.84-0.99) | 0.86 (0.79-0.93) |  | 19.2 % | 0.85 (0.77-0.93) | 0.79 (0.72-0.86) |
| Sub-Saharan Africa | 23.7 % | 0.82 (0.75-0.90) | 0.74 (0.68-0.81) |  | 14.6 % | 0.72 (0.65-0.79) | 0.63 (0.58-0.70) |

|  | **Inadequate GWG in normal weight women using original calculation (main results)** | | |  | **Inadequate GWG in normal weight women using  INTERGROWTH-21 cut-offs^1^** | | |
| --- | --- | --- | --- | --- | --- | --- | --- |
|  | **Prevalence** | **OR (95 % CI)** | **OR (95 % CI)** |  | **Prevalence** | **OR (95 % CI)** | **OR (95 % CI)** |
| **Birth region** |  | ***Basic adjustment***^2^ | ***Basic adjustment***^2^ ***+ education*** |  |  | ***Basic adjustmen***^2^ | ***Basic adjustment***^2^ ***+ education*** |
| Sweden | 21.9 % | Reference | Reference |  | 9.0 % | Reference | Reference |
| Central Europe, Eastern Europe and Central Asia | 17.1 % | 0.85 (0.80-0.91) | 0.83 (0.78-0.89) |  | 7.4 % | 0.89 (0.82-0.97) | 0.86 (0.79-0.93) |
| High income countries | 26.0 % | 1.14 (1.06-1.22) | 1.14 (1.06-1.22) |  | 10.8 % | 1.15 (1.04-1.26) | 1.15 (1.05-1.27) |
| Latin America and Caribbean | 28.2 % | 1.27 (1.09-1.47) | 1.25 (1.07-1.45) |  | 13.0 % | 1.42 (1.18-1.72) | 1.37 (1.14-1.66) |
| North Africa and Middle East | 22.8 % | 1.18 (1.12-1.24) | 1.10 (1.04-1.16) |  | 10.8 % | 1.30 (1.21-1.39) | 1.16 (1.09-1.25) |
| South Asia | 31.8 % | 1.68 (1.48-1.91) | 1.65 (1.45-1.87) |  | 17.7 % | 2.16 (1.87-2.49) | 2.11 (1.82-2.43) |
| Southeast Asia and East Asia | 22.7 % | 0.98 (0.90-1.08) | 0.92 (0.84-1.00) |  | 10.0 % | 1.07 (0.95-1.20) | 0.95 (0.84-1.07) |
| Sub-Saharan Africa | 41.1 % | 2.26 (2.10-2.44) | 1.97 (1.82-2.14) |  | 24.1 % | 2.92 (2.70-3.17) | 2.34 (2.15-2.56) |

^1^ GWG was classified according to the INTERGROWTH-21 weigh gain standards (< 1 in z-score = inadequate GWG; > 1 in z-score = excessive GWG).

^2^ Basic adjustments in the analyses were age, parity and gestational age at first antenatal care visit.
