# Supplementary material for: Body mass index and gestational weight gain in migrant women by birth regions compared with Swedish-born women: A registry linkage study of 0.5 million pregnancies
Source: PLoS One. 2020 Oct 29;15(10):e0241319. doi: 10.1371/journal.pone.0241319 (PMC7595374; doi:10.1371/journal.pone.0241319)
Supplement: S11 Table — (DOCX) [file pone.0241319.s014.docx]

**S11 Table.** Differences in body mass index (kg/m^2^) in the first trimester of pregnancy by birth regions as calculated by means of quantile regression.

|  | **Basic adjustment**^1^ | | |  | **Basic adjustment**^1^ **+ education** | | |
| --- | --- | --- | --- | --- | --- | --- | --- |
| **Birth region** | **10^th^ percentile** | **50^th^ percentile** | **90^th^ percentile** |  | **10^th^ percentile** | **50^th^ percentile** | **90^th^ percentile** |
| Sweden | Reference | Reference | Reference |  | Reference | Reference | Reference |
| Central Europe, Eastern Europe and Central Asia | -0.35  (-0.40, -0.30) | -0.45  (-0.51, -0.39) | -1.16  (-1.33, -1.00) |  | -0.38  (-0.43, -0.33) | -0.62  (-0.68, -0.56) | -1.31  (-1.47, -1.15) |
| High income countries | -0.35  (-0.41, -0.28) | -0.45  (-0.53, -0.37) | -0.24  (-0.45, -0.03) |  | -0.30  (-0.37, -0.24) | -0.31  (-0.39, -0.23) | -0.11  (-0.32, 0.09) |
| Latin America and Caribbean | 0.30  (0.17, 0.43) | 0.58  (0.41, 0.74) | 0.03  (-0.41, 0.47) |  | 0.22  (0.09, 0.35) | 0.35  (0.19, 0.51) | -0.29  (-0.71, 0.14) |
| North Africa and Middle East | 0.54  (0.50, 0.58) | 1.04  (0.99, 1.10) | 0.35  (0.21, 0.49) |  | 0.47  (0.42, 0.51) | 0.70  (0.65, 0.75) | -0.21  (-0.35, -0.07) |
| South Asia | -0.23  (-0.35, -0.12) | 0.49  (0.34, 0.63) | -0.48  (-0.86, -0.10) |  | -0.24  (-0.35, -0.12) | 0.49  (0.35, 0.63) | 0.08  (-0.29, 0.45) |
| Southeast Asia and East Asia | -1.22  (-1.30, -1.13) | -1.66  (-1.77, -1.56) | -3.41  (-3.68, -3.13) |  | -1.33  (-1.42, -1.25) | -2.02  (-2.12, -1.92) | -3.76  (-4.02, -3.49) |
| Sub-Saharan Africa | -0.10  (-0.17, -0.04) | 1.51  (1.43, 1.59) | 1.51  (1.29, 1.73) |  | -0.24  (-0.30, -0.17) | 0.84  (0.75, 0.92) | 0.13  (-0.08, 0.35) |

^1^ Basic adjustments in the analyses were age, parity and gestational age at first antenatal care visit.
