# Supplementary material for: Body mass index and gestational weight gain in migrant women by birth regions compared with Swedish-born women: A registry linkage study of 0.5 million pregnancies
Source: PLoS One. 2020 Oct 29;15(10):e0241319. doi: 10.1371/journal.pone.0241319 (PMC7595374; doi:10.1371/journal.pone.0241319)
Supplement: S12 Table — (DOCX) [file pone.0241319.s015.docx]

**S12 Table.** Differences in gestational weight gain (kg per week) by birth regions as calculated by means of quantile regression.

|  | **Basic adjustment^1^** | | |  | **Basic adjustment^1^ + education** | | |
| --- | --- | --- | --- | --- | --- | --- | --- |
| **Birth region** | **10^th^ percentile** | **50^th^ percentile** | **90^th^ percentile** |  | **10^th^ percentile** | **50^th^ percentile** | **90^th^ percentile** |
| Sweden | Reference | Reference | Reference |  | Reference | Reference | Reference |
| Central Europe, Eastern Europe and Central Asia | 0.026  (0.021, 0.032) | 0.032  (0.028, 0.035) | 0.044  (0.038, 0.051) |  | 0.032  (0.026, 0.037) | 0.034  (0.030, 0.037) | 0.040  (0.034, 0.047) |
| High income countries | -0.009  (-0.016, -0.001) | -0.017  (-0.022, -0.012) | -0.026  (-0.035, -0.017) |  | -0.013  (-0.020, -0.006) | -0.017  (-0.022, -0.012) | -0.022  (-0.030, -0.013) |
| Latin America and Caribbean | -0.040  (-0.054, -0.025 | -0.033  (-0.043, -0.023) | -0.042  (-0.060, -0.025) |  | -0.032  (-0.047, -0.018) | -0.033  (-0.043, -0.023) | -0.047  (-0.064, -0.029) |
| North Africa and Middle East | -0.030  (-0.035, -0.026) | -0.011  (-0.014, -0.007) | 0.007  (0.002, 0.013) |  | -0.009  (-0.014, -0.004) | -0.004  (-0.008, -0.001) | 0.003  (-0.003, 0.008) |
| South Asia | -0.060  (-0.073, -0.048) | -0.061  (-0.069, -0.052) | -0.063  (-0.078, -0.047) |  | -0.064  (-0.077, -0.051) | -0.057  (-0.065, -0.048) | -0.056  (-0.071, -0.041) |
| Southeast Asia and East Asia | 0.010  (0.000, 0.019) | -0.007  (-0.013, -0.000) | -0.043  (-0.055, -0.032) |  | 0.020  (0.010, 0.029) | -0.002  (-0.008, 0.005) | -0.043  (-0.055, -0.032) |
| Sub-Saharan Africa | -0.167  (-0.174, -0.160) | -0.120  (-0.125, -0.116) | -0.101  (-0.109, -0.093) |  | -0.118  (-0.125, -0.111) | -0.106  (-0.111, -0.101) | -0.110  (-0.119, -0.101) |

^1^ Basic adjustments in the analyses were age, parity and gestational age at first antenatal care visit.
