# Supplementary material for: Body mass index and gestational weight gain in migrant women by birth regions compared with Swedish-born women: A registry linkage study of 0.5 million pregnancies
Source: PLoS One. 2020 Oct 29;15(10):e0241319. doi: 10.1371/journal.pone.0241319 (PMC7595374; doi:10.1371/journal.pone.0241319)
Supplement: S13 Table — (DOCX) [file pone.0241319.s016.docx]

**S13 Table.** Comparing the odds ratios of obesity, underweight as well as excessive and inadequate gestational weight gain (GWG) by birth regions, with additional adjustments for the use of an interpreter in maternity care (yes vs. no) as a proxy for acculturation^1^.

|  | **Obesity** | |  | **Underweight** | |
| --- | --- | --- | --- | --- | --- |
|  | **OR (95 % CI)** | **OR (95 % CI)** |  | **OR (95 % CI)** | **OR (95 % CI)** |
| **Birth region** | ***Basic adjustmen^1^ + education*** | ***Basic adjustment^1^ + education +***  ***use of interpreter*** |  | ***Basic adjustment^1^ + education*** | ***Basic adjustment^1^ + education +***  ***use of interpreter*** |
| Sweden | Reference | Reference |  | Reference | Reference |
| Central Europe, Eastern Europe and Central Asia | 0.66 (0.64-0.70) | 0.66 (0.63-0.69) |  | 1.47 (1.37-1.58) | 1.42 (1.31-1.53) |
| High income countries | 0.99 (0.94-1.05) | 0.99 (0.94-1.05) |  | 1.43 (1.30-1.59) | 1.43 (1.28-1.58) |
| Latin America and Caribbean | 1.04 (0.93-1.15) | 1.03 (0.92-1.15) |  | 1.14 (0.90-1.45) | 1.04 (0.80-1.35) |
| North Africa and Middle East | 1.08 (1.04-1.12) | 1.06 (1.02-1.10) |  | 1.00 (0.93-1.09) | 0.94 (0.86-1.02) |
| South Asia | 1.10 (1.00-1.21) | 1.11 (1.00-1.23) |  | 2.00 (1.71-2.33) | 2.01 (1.72-2.36) |
| Southeast Asia and East Asia | 0.23 (0.21-0.26) | 0.23 (0.20-0.25) |  | 3.27 (3.01-3.55) | 3.21 (2.95-3.50) |
| Sub-Saharan Africa | 1.32 (1.25-1.38) | 1.30 (1.23-1.37) |  | 2.40 (2.20-2.62) | 2.24 (2.04-2.46) |
|  | **Excessive GWG** | |  | **Inadequate GWG** | |
|  | **OR (95 % CI)** | **OR (95 % CI)** |  | **OR (95 % CI)** | **OR (95 % CI)** |
| **Birth region** | ***Basic adjustment^1^ + education*** | ***Basic adjustment^1^ + education +***  ***use of interpreter*** |  | ***Basic adjustment^1^ + education*** | ***Basic adjustment^1^ + education +***  ***use of interpreter*** |
| Sweden | Reference | Reference |  | Reference | Reference |
| Central Europe, Eastern Europe and Central Asia | 1.16 (1.12-1.21) | 1.18 (1.13-1.27) |  | 0.85 (0.81-0.90) | 0.84 (0.79-0.89) |
| High income countries | 0.86 (0.82-0.91) | 0.87 (0.83-0.92) |  | 1.12 (1.06-1.20) | 1.15 (1.08-1.22) |
| Latin America and Caribbean | 0.89 (0.80-0.99) | 0.89 (0.80-0.99) |  | 1.21 (1.07-1.37) | 1.16 (1.01-1.32) |
| North Africa and Middle East | 1.11 (1.08-1.15) | 1.13 (1.09-1.18) |  | 0.99 (0.95-1.04) | 0.95 (0.91-1.00) |
| South Asia | 0.87 (0.79-0.95) | 0.89 (0.80-0.98) |  | 1.42 (1.28-1.58) | 1.43 (1.28-1.59) |
| Southeast Asia and East Asia | 0.62 (0.58-0.66) | 0.60 (0.56-0.64) |  | 0.92 (0.85-1.00) | 0.89 (0.82-0.97) |
| Sub-Saharan Africa | 0.62 (0.59-0.66) | 0.63 (0.59-0.67) |  | 1.73 (1.63-1.83) | 1.68 (1.58-1.78) |

^1^ Basic adjustments in the analyses were age, parity and gestational age at first antenatal care visit.
